# Age-related changes in the physical properties, cross-linking, and glycation of collagen from mouse tail tendon

**DOI:** 10.1074/jbc.RA119.011031

**Published:** 2020-05-07

**Authors:** Melanie Stammers, Irina M. Ivanova, Izabella S. Niewczas, Anne Segonds-Pichon, Matthew Streeter, David A. Spiegel, Jonathan Clark

**Affiliations:** 1 Babraham Institute, Cambridge, United Kingdom; 2 John Innes Centre, Norwich, United Kingdom; 3 Department of Chemistry, Yale University, New Haven, Connecticut, USA

**Keywords:** tendon, collagen, cross-links, lysine glycation, aging, physical strain, chemistry, diabetes, mechanical stress, connective tissue, tendon, protein cross-linking

## Abstract

Collagen is a structural protein whose internal cross-linking critically determines the properties and functions of connective tissue. Knowing how the cross-linking of collagen changes with age is key to understanding why the mechanical properties of tissues change over a lifetime. The current scientific consensus is that collagen cross-linking increases with age and that this increase leads to tendon stiffening. Here, we show that this view should be reconsidered. Using MS-based analyses, we demonstrated that during aging of healthy C57BL/6 mice, the overall levels of collagen cross-linking in tail tendon decreased with age. However, the levels of lysine glycation in collagen, which is not considered a cross-link, increased dramatically with age. We found that in 16-week-old diabetic db/db mice, glycation reaches levels similar to those observed in 98-week-old C57BL/6 mice, while the other cross-links typical of tendon collagen either decreased or remained the same as those observed in 20-week-old WT mice. These results, combined with findings from mechanical testing of tendons from these mice, indicate that overall collagen cross-linking in mouse tendon decreases with age. Our findings also reveal that lysine glycation appears to be an important factor that contributes to tendon stiffening with age and in diabetes.

The literature surrounding the mechanical and chemical properties of collagen and changes which occur with age is extensive. The general consensus is that as collagen ages there is an increase in the stiffness with loss of elasticity and that this is due to an increase in covalent intermolecular cross-linking between collagen molecules which develops with age (1). In this paper we challenge the simplicity of this conclusion.

The collagen cross-links can be divided into two groups, those that are of enzymatic origin and those that form through purely chemical reactions with reactive molecules perfusing the tissues. The enzymatically derived cross-links that are first formed can be analyzed after reduction with sodium borohydride (2, 3) and the reduced products measured as dihydroxy-lysino-norleucine (DHLNL), hydroxy-lysino-norleucine (HLNL), and lysino-norleucine (LNL) (structures shown in Fig. 1). Another collagen cross-link, histidine-hydroxymerodesmosine (HHMD), is commonly found during the analysis of reduced samples (4) by MS. While there has been disagreement (5, 6) with respect to the exact chemical structure that the analyzed compound represents within collagen, for the purposes of this publication it can be considered an indicator of aldol cross-links present in collagen before analysis.

**Figure 1.**
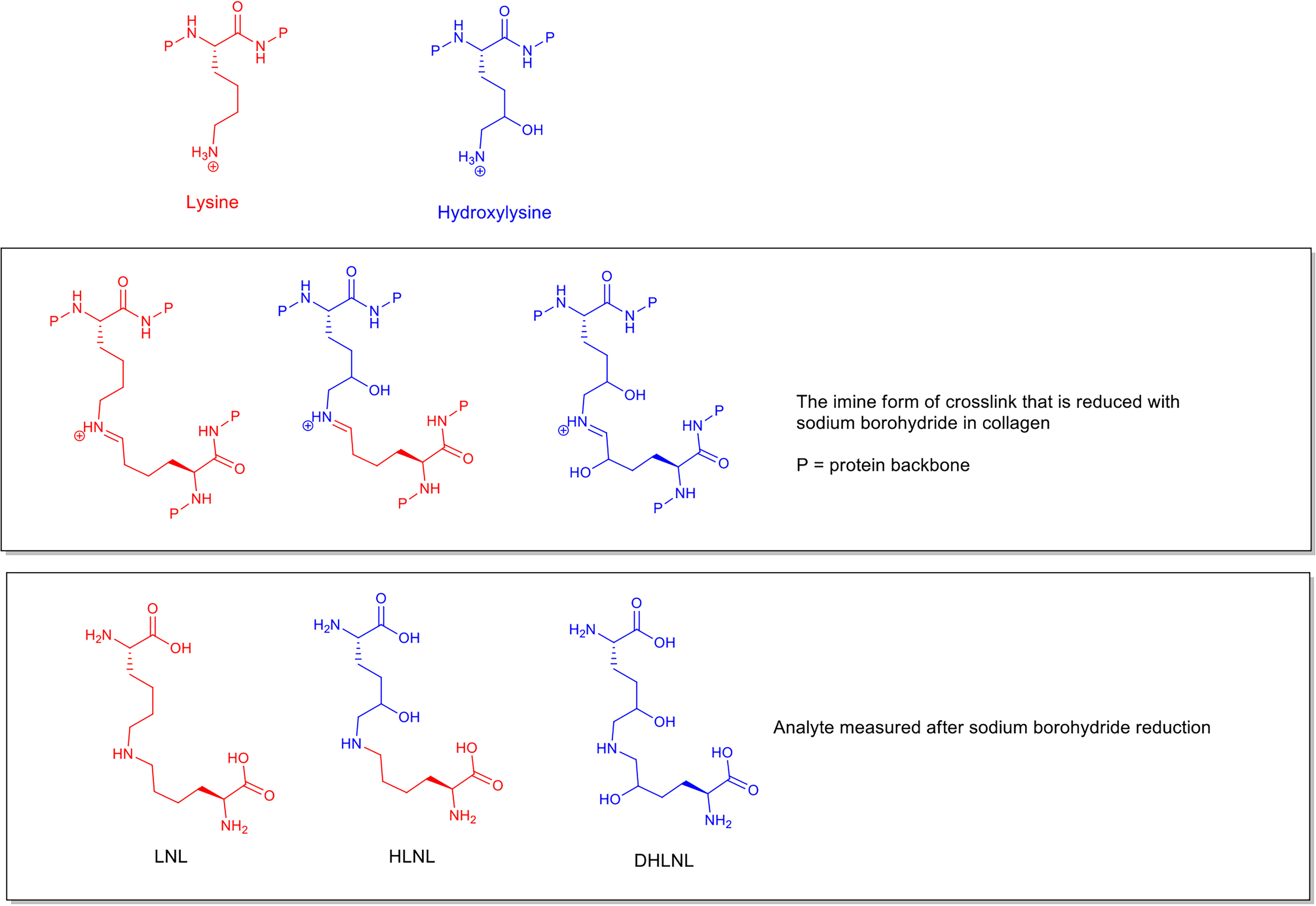
**Structures of LNL, HLNL, and DHLNL analyzed by MS after treatment with sodium borohydride.** The relationship to lysine and hydroxylysine and the intermediate imine cross-link structures found in collagen are also shown.

These cross-links are early structures in the enzymatic cross-linking process and are often described as immature cross-links. The immature cross-links can go on to form irreversible cross-links (1) (pyridinolines), often described in the literature as “mature” cross-links, through further reactions. It has been noted in numerous papers that the number of immature cross-links per mole of collagen decreases with age and that the increase in mature pyridinoline cross-links does not seem to match this decrease (7–9), implying that the loss of immature cross-links cannot be entirely explained by their conversion into mature cross-links. In papers describing mature cross-link formation, the emphasis is usually on the increase in mature cross-links, and this increase is correlated to an increase in stiffness of the tissue; however, this overlooks the fact that there is potentially an overall decrease in cross-linking with age from the loss of the immature cross-links. The paradox here is that if total cross-linking is decreasing with age, then how is it that the tissues get stiffer?

Cross-links formed through nonenzymatic processes involving sugars are referred to as advanced glycation end products (AGEs), and in this paper, we include them when referring to mature cross-links. These are formed through the reaction of sugars or products of sugar metabolism with collagen, which then react further to create cross-links. The AGE cross-links generally considered to be most important are glucosepane and pentosidine (9, 10). A significant proportion of the literature has focused on increased AGE cross-linking, particularly glucosepane, as a potential cause of stiffening in aging tendon. This has been concluded through correlations of measured AGE cross-link levels with the mechanical testing of diabetic tissue and of collagen incubated *in vitro* in the presence of sugars. However, these studies measured the absolute glucosepane concentration not as a proportion of the total collagen content but usually as a relative increase in signal (11) or as a proportion of the extracted insoluble collagen content (12). Here we measured the absolute glucosepane content as a proportion of the total collagen content of tendon in addition to the pentosidine content and the glycation products of lysine and hydroxylysine. Although increases in glucosepane with age have been demonstrated, without knowing the absolute amount relative to the total collagen content it is impossible to assess if glucosepane cross-linking is an important factor in the stiffening of normal healthy tendon during aging and what the impact of other AGEs might be.

In this study we chose to use mouse tail tendon because it is primarily collagen I and the impact of altered cross-linking on the mechanical properties can be readily tested. We used tendon from WT C57BL/6 mice because they have been shown not to develop diabetes (13) and so represent a model of healthy aging. We studied both the mechanical and chemical changes across a population of mice from 8 weeks to 100 weeks old. In order to remove as many population variables as possible and show what happens in the normal, healthy aging process, a cohort of C57BL/6 mice housed under highly controlled conditions of diet and environment were used.

The aim of the study described here was to clarify the quantitative changes in cross-linking that occur in normal healthy mouse tail tendon with age and identify which cross-links are likely to give rise to the altered physical properties with age.

## Results

### Confirmation of basic mouse model parameters

Blood glucose levels were found to be controlled well across all age groups under fasting and nonfasting conditions (Fig. S1, *a* and *b*).

The approximate rate of collagen synthesis in tendon at 33, 63, and 88 weeks of age was determined by feeding [^13^C_6_]lysine for 28 days and then measuring the incorporation of the label into the collagen within the tissues. The rate of incorporation of [^13^C_6_]lysine was found to be between 0.04% and 0.06% collagen lysine content per day in tail tendon, showing little change with age (Fig. S1*c*).

### Profiling changes in the physical properties of tail tendon with age

**Figure 2.**
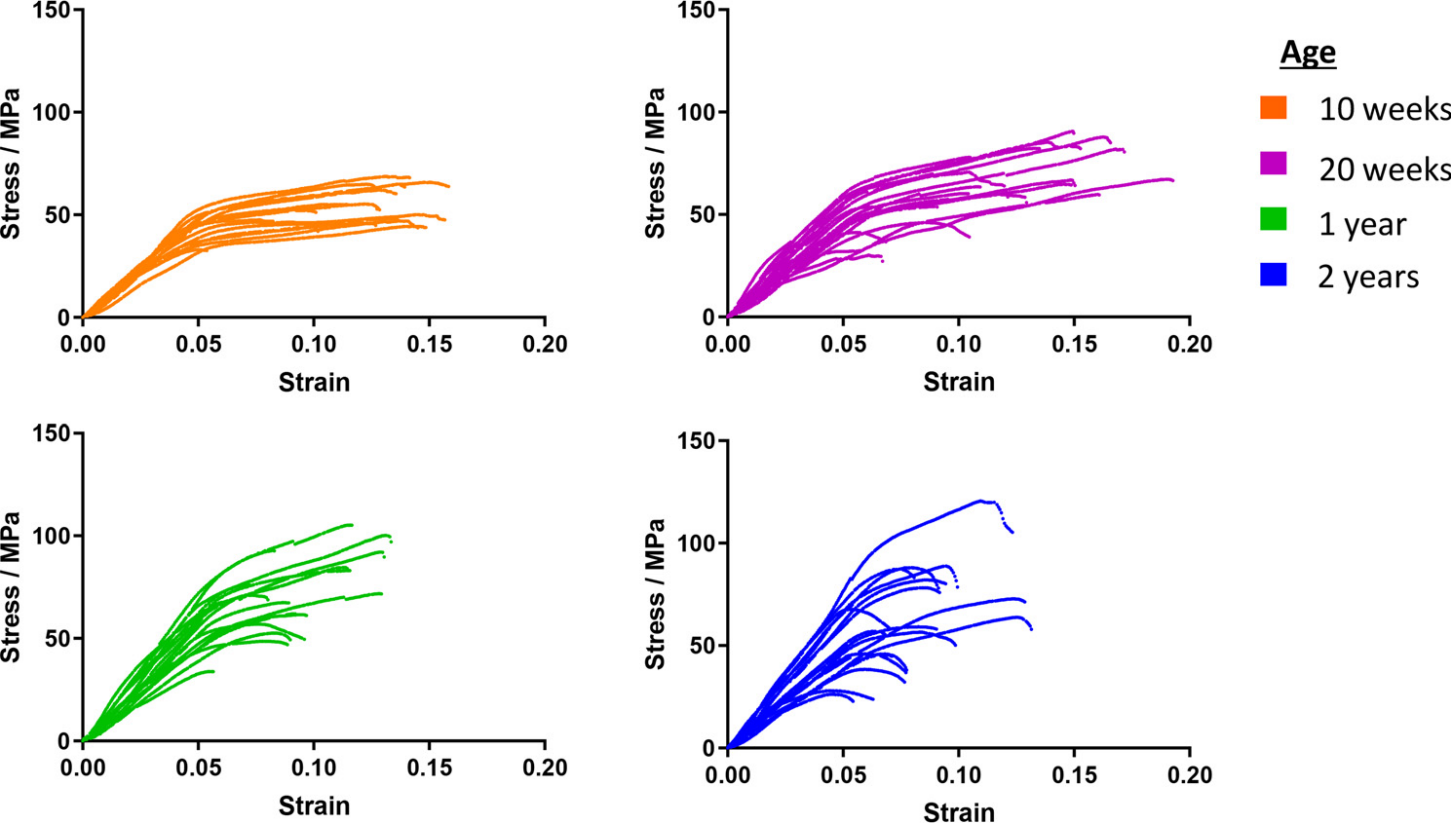
**Plots showing representative stress-strain profiles from multiple tendon fibers at four ages.** Five mice from each age group were sampled.

The stress-strain profiles for multiple tail tendons from 5 mice with a range of ages were measured using a tensile stress stage (“stress” is defined as force/cross-sectional area; “strain” is defined as extension/initial length). There are 4 tendons for each vertebral bone in the tail, which run from the base of the tail to each bone insertion point in four bundles of tendons. The individual tendons were carefully dissected out from the bundles for use. Individual tendons were stretched to the breaking point at a rate of 1 mm/min and the stress measured every 0.5 s. The stress-strain profiles (Fig. 2) for the fibers from younger animals were more homogeneous across the population than those from older animals. Tendon from 10-week-old mice showed an initial stretching phase followed by a long plastic extension phase before breaking. The stress-strain profiles of tendons from older animals had a much greater spread, with some fibers still showing a plastic extension phase and others breaking earlier at a lower strain with only a modest plastic extension phase. With increasing age, a greater proportion of the tendons showed an increase in the gradient of the initial stretching phase; that is, the stiffness (Young's modulus) of the tendons increased. The initial stretching phase in animals of all ages extended to about 5% strain before transitioning into the plastic phase. Young adult tendons in the 20-week-old group frequently reached a greater strain before breaking than either younger tendons or tendons more than 1 year old. The picture of changes seen here is more subtle than the impression usually presented in the literature, where a simplified schematic of increasing stiffness of tendon with age is shown.

### Cross-link profiles with age

The cross-links present and collagen content were measured by HPLC-MS using tissue processing and MS methods developed for this project. It was of particular concern to us to remove reactive soluble compounds released from cell lysis, such as ribose-5-phosphate, which are known to be highly reactive glycating molecules. To do this we used a continuous-flow system to wash the soluble components from the samples. We confirmed that the processing method for removal of soluble components did not impact the proportions of analytes under study by comparing the levels in tendon immediately after dissection without processing and after processing. In addition to this, a novel method for the analysis of the AGE cross-link glucosepane by MS was developed where the acid hydrolysis products of both the endogenous and synthetic ^13^C_5_-labeled glucosepane used to spike the sample before hydrolysis were measured.

**Figure 3.**
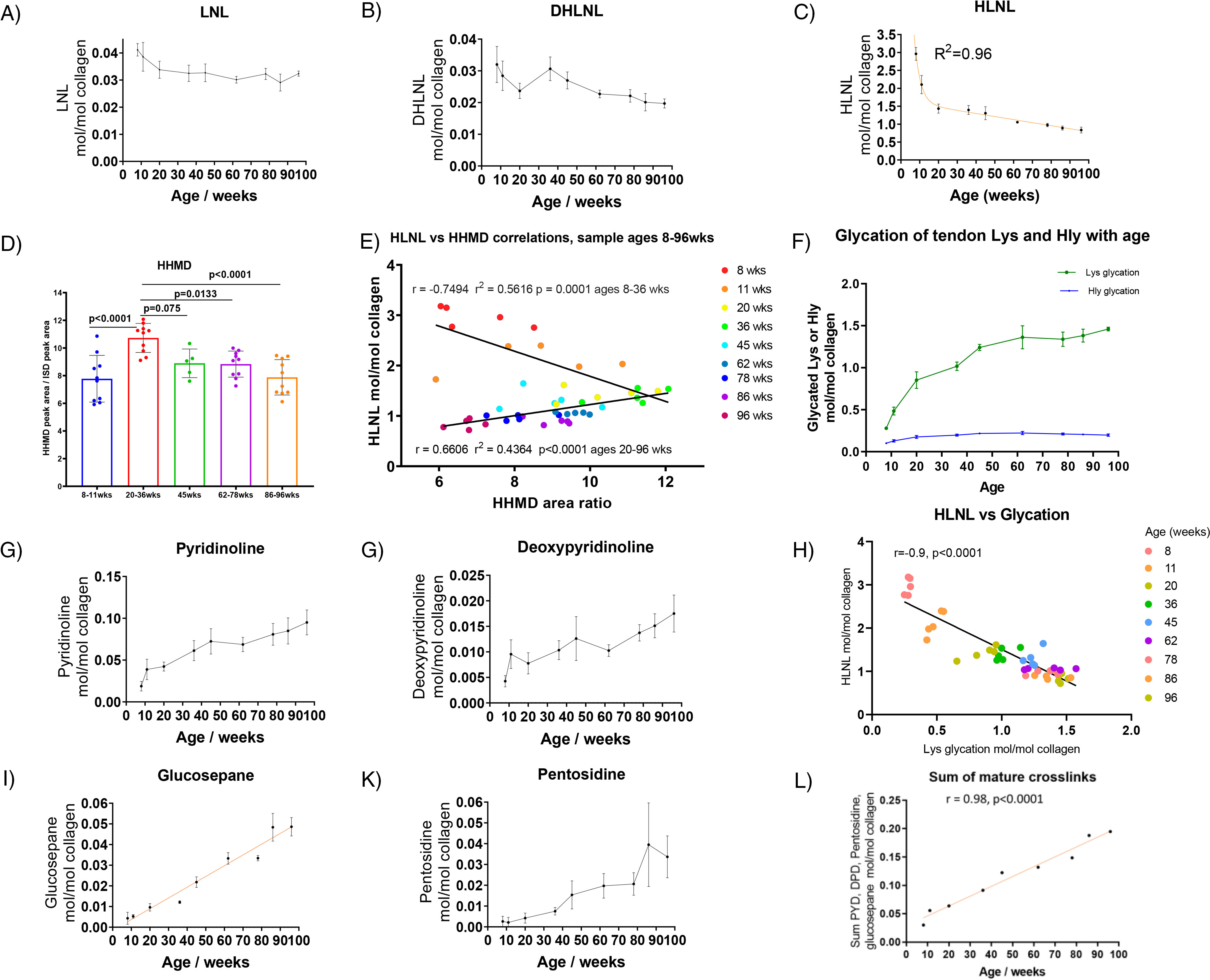
*A*, *B*, and *C*, levels of the immature cross-links LNL, DHLNL, and HLNL with age in the tendon fibers of C57BL/6 mice (*n* = 5 per age group, in duplicate). Statistical analysis: nonlinear regression fit shown in the HLNL plot (mean ± S.D.). *D*, Change in the aldol product HHMD with age. Statistical analysis: ANOVA followed by Tukey's multiple-comparison tests (mean ± S.D., *n* = 5 to 10). *E*, Correlations between HLNL and HHMD with age. Statistical analysis: linear regression. *F*, levels of glycation products with age (mean ± S.D., *n* = 5, in duplicate). Lys glycation is shown in green; Hly glycation is shown in blue. *G*, *H*, *J*, and *K*, development of irreversible cross-links with age in the tendon fibers of C57BL/6 mice (*n* = 5, in duplicate). Statistical analysis (*J*): linear regression fit (mean ± S.D., *n* = 5, in duplicate). *I*, correlation between HLNL levels and glycation levels. Statistical analysis: linear regression. *L*, sum of PYD, DPD, pentosidine, and glucosepane collagen values (mol/mol) plotted against age. Statistical analysis: linear regression.

Fig. 3 shows changes in the cross-links found in mouse tail tendon with age. The most abundant cross-link at all ages was HLNL, and the greatest change with age occurred in the level of this cross-link. A large drop in HLNL occurred up to 20 weeks when the animal was maturing, decreasing from 3 cross-links per collagen molecule at 8 weeks to 1.5 cross-links per collagen molecule at 20 weeks. HLNL levels continued to decrease more gradually after the animal had matured, falling to ∼0.85 cross-links per collagen molecule at 96 weeks.

While we could not quantify the absolute amount of HHMD due to the lack of a synthetic standard, we were able to measure the relative change in the mass spectrometer detector response. HHMD can be seen to increase significantly up to 20–36 weeks and then significantly decrease as the animals age (Fig. 3*D*). Both HHMD and HLNL are formed from aldehydes generated by the activity of lysyl oxidase. The aldehydes could also be produced from the hydrolysis of HLNL, and because we saw a large drop in HLNL up to 20 weeks, we looked at whether a correlation between HLNL and HHMD could be seen. There were clearly two phases in the data shown in the correlation plots between HLNL and HHMD (Fig. 3*E*). In the first phase, between 8 and 36 weeks, it can be seen that as HLNL decreases, HHMD increases. In the second phase, from 36 to 96 weeks, it can be seen that both HLNL and HHMD decline with age. These data suggest that as the HLNL levels drop rapidly in early life, the aldehyde generated undergoes an aldol reaction and HHMD formation faster than any alternative aldehyde or aldol removal process. In later life, the rate of formation of aldol adducts is then lower than the rate of removal, and so both the levels of HLNL and HHMD are seen to decline.

In an attempt to estimate the possible level of HHMD, we made an assumption that the HLNL change between the ages of 11 weeks and 20 weeks is converted into the measured change in HHMD based on the correlation seen. With this assumption, the level of HHMD converts to about 1.1 HHMD per collagen molecule at 20 weeks and 0.6 to 0.8 HHMD per collagen molecule at 96 weeks (the conversion factor is ∼10 HHMD area ratio units to 1 HHMD molecule in Fig. 3*D*). This level of HHMD is broadly consistent with reported levels of the compound found in tendon using tritium labeling and ion-exchange chromatography (14).

No single mature cross-link type increased dramatically with age; however, as a group, mature cross-links contributed to a modest increase in overall number of cross-links per collagen molecule (Fig. 3*L*). The increase in mature cross-links equated to an increase from 1 cross-link per 33 collagen molecules at 10 weeks to 1 cross-link in 5 collagen molecules at 96 weeks. This increase in mature cross-links was 3 times less than the drop in HLNL between 20 weeks and 96 weeks (Fig. 3, *C* and *L*). The sum of mature and immature cross-links at 96 weeks was ∼1 cross-link per collagen molecule excluding the HHMD contribution.

### Changes in glycation with age

Over the lifetime of the animals, there was only a modest increase in AGE cross-links; however, in contrast with this, the increase in lysine glycation was a major change (Fig. 3*F*). The glycation of lysine is the direct addition of a monosaccharide, typically glucose, to the side chain nitrogen of lysine and is not a cross-link. Cross-link formation would require further reaction with another residue in the collagen structure. The addition reaction can also happen on the side chain of hydroxylysine.

By 60 weeks of age, the glycation of tendon collagen lysine reached 1.5 per collagen molecule, the glycation of hydroxylysine being much lower at about 0.2 per molecule of collagen. The level of lysine glycation appears to plateau after 50 weeks of age. Increased levels of glycation appear to correlate with decreased HLNL levels, although both were changing rapidly during the growth phase, and the changes may not be causally linked (Fig. 3*I*). It has been reported that glycation occurs at the same site as the lysyl oxidase-mediated cross-links (15); however, in that report only glycation of Hly was seen. In our study, we saw glycation of Lys rather than Hly as the major adduct.

### Diabetic tendon

**Figure 4.**
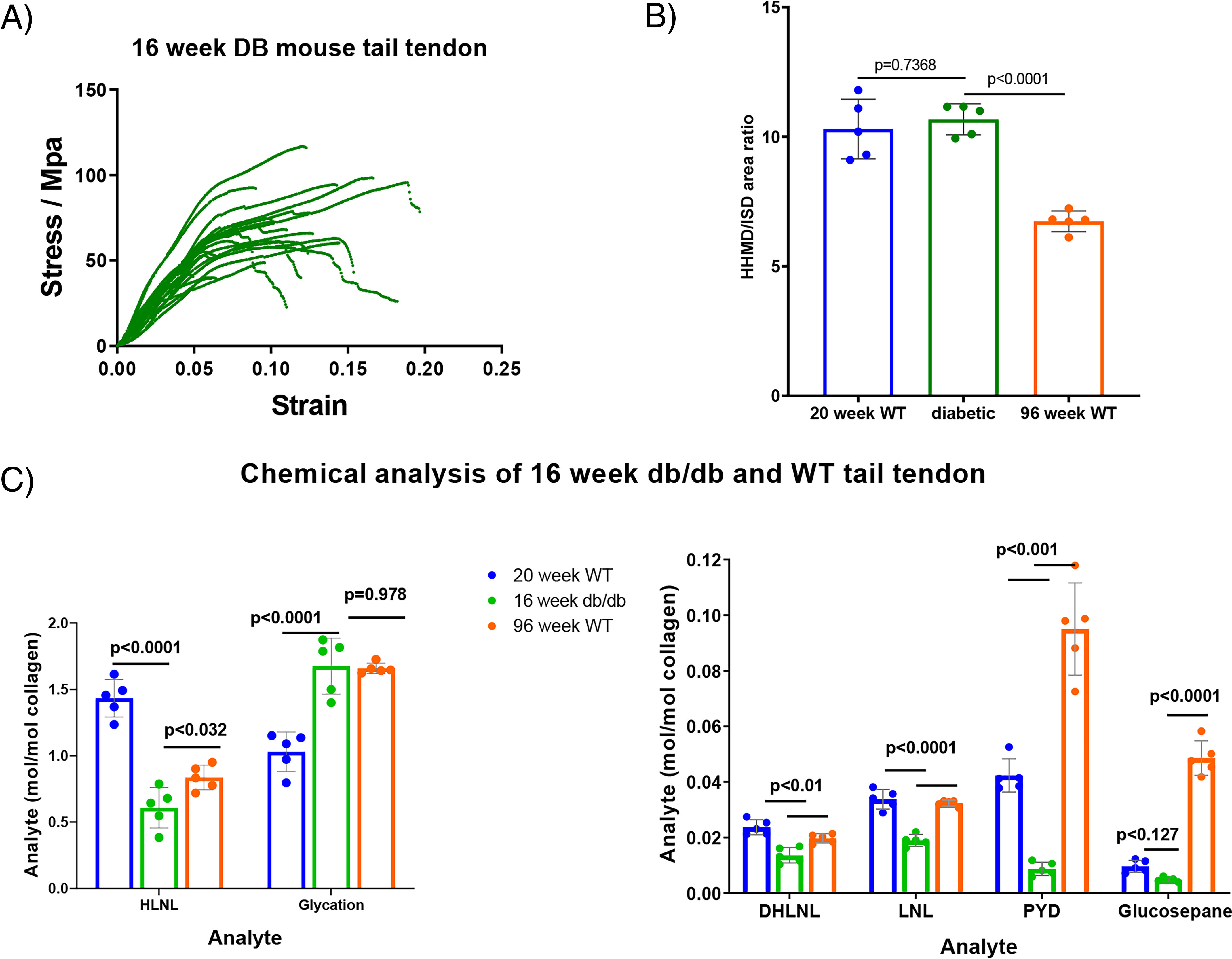
*A*, stress strain plot showing representative tail tendon fibers from five db/db mice. The number of fibers is limited in this plot so that the shape of the lines can be seen more easily. *B*, HHMD analysis of db/db mouse tail tendon fibers compared with data from WT mice 20 and 96 weeks old (mean ± S.D., *n* = 5, in duplicate). Statistical analysis: ANOVA followed by Tukey's multiple-comparison tests. *C*, chemical analysis of db/db mouse tail tendon fibers compared with data from WT mice 20 and 96 weeks old (mean ± S.D., *n* = 5, in duplicate). Statistical analysis: ANOVA followed by Tukey's multiple-comparison tests.

With the changes seen in collagen lysine side chain glycation in healthy WT C57BL/6 mice, we decided to examine the levels in diabetic mice. Tail tendon samples from 16-week-old JAX™ db/db mice (Charles River) on the C57BLKS/J background were obtained. These diabetic mice have a point mutation in the Lepr gene and are used as an animal model for diabetes research. The tendon fibers were subjected to physical and chemical analysis as described above, and the results are shown in Fig. 4*A* and Fig. S2. The spread and stiffness observed in the break test profiles of tendon from 16-week-old db/db mice resembled those seen in tendon from older WT animals, while the plastic extension phase often looked more like that in tendon from younger animals (less than 1 year). Kinks and wobbles in the profiles of the db/db tendon can be seen in the traces and were more prevalent than in WT tendon.

Chemical analysis showed that levels of all forms of detectable cross-link for the diabetic animals, apart from HHMD, were lower than the levels found in both 20- and 96-week-old WT animals (Fig. 4, *B* and *C*). The HHMD level was not significantly different between 20-week-old WT and 16-week-old db/db tendon. The HLNL level in 16-week-old db/db tendon was found to be ∼0.6 HLNL bonds per collagen molecule, which is much lower than the 1.5 HLNL bonds per collagen molecule found in 20-week-old WT tendon. The level of glycation in the 16-week-old db/db tendon was comparable to that in 96-week-old WT mice and much higher than that seen in 20-week-old WT mice. The levels of irreversible cross-links in the db/db mice, both the mature and AGE classes, were lower than those in 20-week-old WT mice.

## Discussion

In the literature on aging collagen, the focus is usually on the increase in mature cross-links formed through mature covalent bonding as the reason for the changing properties observed. What we saw here shows that the situation is more complex than this and that both the loss of reversible cross-links and increase in lysine glycation are important changes to consider in the overall picture of how tendon collagen functions.

The often-stated view that tendon stiffens with age due to an increase in cross-linking does not fit the entire data set shown here, and in light of the db/db data, it appears that the mechanical changes do not have to be linked to an increase in AGEs such as glucosepane or pentosidine. With the marked decrease in cross-linking in db/db mice, it might be expected that the tendons would be less stiff than those of older WT mice; however, the stiffness of the db/db tendons is similar to that in older WT mice. The one clear factor that could give rise to the observed stiffness of db/db tendon is the increased glycation of lysine residues, which is similar to that found in tendon from 98-week-old mice. It is apparent from the kinks and wobbles in the db/db stress-strain profiles that there are faults in the tendon structure. It is likely that these faults are due to the lower levels of cross-linking, explaining why the diabetic tendon is often unable to maintain structure under stress.

While tendon does generally stiffen with age, another important change is the loss of the plastic extension phase. It seems likely from the results presented here that the loss of the plastic phase is due to a decrease in immature cross-links with an increase in mature cross-linking, while the stiffening is caused by both the glycation of lysine and mature cross-link formation. The mechanisms by which cross-links contribute to these properties and a demonstration of the involvement of glycated lysine in tendon functioning are the subject of an accompanying paper (16).

In conclusion, tendon stiffening and the loss of the plastic phase in extension are important mechanical changes with age and diabetes observed in this study. Defining the parameters for healthy aging requires an understanding of the underlying chemical changes that underpin these mechanical changes. Here we have shown that lysine glycation products are likely to play a significant role in these mechanical changes as well as the altered distribution of immature and mature enzymatic cross-links.

## Materials and methods

### Animal procedures

#### 

##### Ethics

Animal experiments were performed according to the UK Animals (Scientific Procedures) Act 1986, license PPL 70/8303, and approved by the Babraham Institute Animal Welfare and Ethics Review Body.

##### Blood glucose measurements

Cohorts of C57BL/6 mice were culled using the standard schedule 1 method of CO_2_ exposure for killing the mice followed by cardiac bleed as the secondary method of confirmation. The glucose level was measured in the blood from the cardiac bleed and from a drop of blood taken from the tail vein after culling. The blood glucose level was measured using S.D. CodeFree blood glucose strips from S.D. Biosensor.

##### Labeling with [^13^C_6_]lysine

Cohorts of C57BL/6 mice were fed on MouseExpress l-[^13^C_6_]lysine (99%)-irradiated mouse feed from CK Isotopes for 28 days. After day 28 the mice were switched to a normal diet for a further 7 days and then culled.

### Sample analysis

#### 

##### Tissue processing

After removal of the skin from isolated tails, tendon was drawn out of the tail under PBS (pH 7.4) by grasping the tip and the base of the tail with forceps. Twisting the forceps holding the tip caused the tail to break, bringing attached tendon fibers with it attached to the vertebral bone. Tendon was isolated sequentially one vertebral bone at a time, working up the tail from the tip to the base. Tendon fibers were detached from the tail vertebrae with a scalpel. Cellular and soluble extracellular components were removed from tail tendon by washing in 3% Triton in a continuous-flow system (∼200 ml/24 h) for 3 days, followed by washing in water for 2–3 days (∼200 ml/24 h).

Of note here is that that the chemistry of the immature cross-links is potentially reversible; any processing that changes chemical environment before reduction could perturb the equilibrium and impact the results (17, 18). For example, when we incubated skin with a 50% glycerol solution in PBS (pH 7.4) for 2 h, we found that after reduction there was a 37% drop in the level of HLNL and a 27% drop in Lys glycation. Fig. S3 shows the impact on tendon collagen of using TES/Tris buffer as commonly used (14) compared with PBS that we used in this work. It can be seen that in TES/Tris buffer, HLNL is 13% lower and the glycated lysine is 61% lower than that obtained in PBS. Both TES and Tris contain functionality similar to glycerol and could act chemically in a similar way.

##### Acid hydrolysis

Tail tendon (wet weight, 16 to 22 mg) suspended in 200 μl PBS (pH 7.4) was reduced by the addition of 10 μl of 10 mg/ml NaBH_4_ in 1 mm NaOH. After incubation for 2 h at room temperature, the tendon was washed 3 times with water and then freeze-dried. Lipid was removed by the addition of 1 ml CHCl_3_/MeOH/H_2_O (100:50:5). After incubation overnight at room temperature, the tendon was washed with a further 1 ml CHCl_3_/MeOH/H_2_O (100:50:5) before air-drying and freeze-drying. [^13^C_5_]Glucosepane internal standard was added and samples freeze-dried. Acid hydrolysis was carried out by incubation overnight at 95°C with 200 μl of 7.4 M HCl. Samples were then dried under a stream of nitrogen gas and resuspended in 400 μl 30% MeCN/0.1% formic acid. After filtering through a 0.22-μm nylon filtration membrane, samples were freeze-dried before resuspension at 10 μg/μl (dry weight) in 30% MeCN/0.1% formic acid. Solutions were then made up at different concentrations with appropriate internal standards for the analysis of the different analytes.

##### HPLC-MS method

Five microliters of solution for analysis was injected onto a Cogent Diamond Hydride column (4 μm, 100 Å, 150 × 2.1 mm). Diamond Hydride columns have been described in the analysis of amino acids (19) and collagen cross-links (20). The method here is a modification of these protocols. A gradient of 100% (acetonitrile, 5% water, 0.1% formic acid, 0.005% TFA) to 100% (water, 0.1% formic acid) was run, the details of which are in Table 1. The flow was passed into an electrospray ionization (ESI) probe of a Micromass Quattro Ultima mass spectrometer, and the fragmentation transitions listed below were monitored (mass spectrometer parameters: source temperature, 120°C; desolvation, temperature 350 °C; cone voltage, 3 kV; capillary voltage, 35 V; collision gas, argon; collision voltage, see Table 2).

**Table 1:** **HPLC gradient solvent profile*^*a*^***

Time (min)	% Solvent A	% Solvent B	% Solvent C	Flow (ml/min)	Curve
0	100	0	0	0.4	1
5	60	40	0	0.4	6
7	10	90	0	0.4	6
9	0	0	100	0.4	1
11	0	0	100	0.4	1
12	100	0	0	0.4	1
20	100	0	0	0.4	1

*^a^* Waters Alliance 2795 HPLC. Solvent A, 95% acetonitrile–5% water with 0.1% formic acid and 0.005% trifluoroacetic acid. Solvent B, 20% methanol–80% water with 0.1% formic acid. Solvent C, water, with 0.1% formic acid. Curve 6 is a linear gradient; curve 1 is a step change to the indicated percent solvent.

**Table 2:** **Mass spectrometer mass transitions**

Molecule	Q1	Q2	Collision energy	Dwell time (s)
Proline	116.07	70.06	15	0.25
d7-Proline	123.11	77.11	15	0.25
Hydroxyproline	132.06	68.05	20	0.25
Lysine	147.11	84.08	20	0.25
d4-Lys	151.14	88.1	20	0.25
Hydroxylysine	163.1	82.06	20	0.25
LNL	276.15	84.08	30	0.2
HLNL	292.18	82.08	30	0.2
cHx-Lys	293.18	84.08	30	0.2
DHLNL	308.18	82.08	35	0.2
cHx-Hly	309.18	82.08	30	0.2
Hx-Lys	311.18	84.08	30	0.2
Hx-[^13^C]Lys std	317.18	84.08	30	0.2
Hx-Hly	327.18	82.08	30	0.2
Pentosidine	379.21	187.1	40	0.2
DPD	413.2	84.08	40	0.3
PYD	429.2	82.08	40	0.3
Hydrolyzed [^13^C]glucosepane (M + 2H^+^ ion)	226.64	84.08	20	0.2
Hydrolyzed glucosepane (M + 2H^+^ ion)	224.28	84.08	20	0.2
HHMD	574.31	156.07	55	0.3
6-Hydroxynorleucine	148.09	102.09	10	0.1
6-D_1_-6-Hydroxynorleucine	149.13	103.1	10	0.1

Glycated lysine and glycated hydroxylysine are known to undergo partial cyclization under the acid hydrolysis conditions. Both the linear (*N*-deoxyhexitolyl-l-lysine (Hx-Lys)) and cyclic (cHx-Lys) forms were measured, and the results were added to give the total glycated lysine and total glycated hydroxylysine values.

##### Calibrations

Amino acid calibration curves were made using commercially available amino acid mix from Sigma Aldrich (A9906, lot SLBR9938V). Calibration curves were constructed using 2,3,3,4,4,5,5-d7-dl-Pro (CK Isotopes) as the internal standard for Pro and Hyp and 4,4,5,5-d4-l-lysine as the internal standard for Lys and Hly.

HLNL, LNL, and DHLNL (Santa Cruz Biotechnology, Inc.) were used for calibration curves with d4-lysine (Sigma Aldrich) as an internal standard. The standards and internal standard (ISD) were used to spike acid-hydrolyzed collagen (50 μg) which had not been reduced for the calibration curve.

The DPD (PolyPeptide Group) was used to construct a calibration curve against d4-lysine as the internal standard. Calibration curves we constructed separately and confirmed for deoxypyridinoline (DPD) and pyridinoline (PYD) with a PYD/DPD HPLC mixture (Quidel Corporation).

Hx-[^13^C_6_]lysine, Hx-[^12^C_6_]lysine, and cHx-[^12^C_6_]lysine were made in-house as standards to enable calibration curves to be constructed. The response of the Hx-[^13^C_6_]lysine ISD was found to be the same as that of Hx-[^12^C_6_]lysine. The response of cHx-[^12^C_6_]lysine was found to be a factor of 1.149 less than that of the Hx-[^13^C_6_]lysine ISD.

d4-l-Lysine was used as an internal standard for HHMD for 50-μg sample injections, and Hx-[^13^C_6_]Lys was used for 400-μg sample injections.

##### Glucosepane analysis

[^13^C_5_]Glucosepane made from [^13^C_5_]ornithine was kindly provided by David Spiegel (Yale), synthesized according to his published methods (21) along with synthetic [^12^C]glucosepane. The usual method for measuring glucosepane is to use extensive proteolytic digests of collagen with a series of enzymes over 2 weeks rather than acid hydrolysis, because glucosepane is not considered to be stable to acid. There are a number of issues with the proteolytic digestion method; one is the time it takes to carry out the digests, which does not lend itself to large numbers of samples, and another is that we found, as others have (11), that one is left with a precipitate which is spun down and discarded. Conceptually the discarding of a precipitate seems to be at odds with the complete measurement of cross-links, because it is possible that this precipitate might contain the cross-links of interest. We found that when glucosepane is hydrolyzed in acid, there is an addition of one molecule of water to the structure by MS. By the inclusion of a ^13^C-labeled glucosepane standard before hydrolysis, we found that glucosepane could be measured reliably after hydrolysis by measurement of this adduct. Experiments were carried out to test the method by using both [^13^C]glucosepane and [^12^C]glucosepane at different concentrations to spike collagen samples prior to hydrolysis. We have not explored how universally applicable this method is for the analysis of glucosepane in tissues other than mouse skin and mouse tendon.

##### HHMD identification and analysis

Parent scans on the mass spectrometer were carried out to look for molecular ions in reduced tendon samples that gave rise to a fragment *m*/*z* of 82, a typical fragmentation product of hydroxylysine residues. An expected ion of 574.3 was found at 9.4 min, which was in the expected retention time range on the HPLC system for a structure of the type proposed by Tanzer *et al.* (4). Fragmentation of the 574.3 ion gave a fragmentation pattern (Fig. S4) consistent with the proposed structure.

We then carried out a comparison of the Lys *m*/*z* 84 and Hly *m*/*z* 82 fragments after NaBH_4_ reduction and the Lys *m*/*z* 85 and Hly *m*/*z* 83 fragments after NaBD_4_ reduction. This experiment was to identify the position of the putative imine bond in the unreduced structure. The results from this experiment are shown in Fig. S5. These data, when extrapolated to the precursor before reduction, show that in the unreduced sample there is Hly, a proportionately small amount of imine from Hly aldehyde, a Lys fragment, and a level of imine from Lys aldehyde similar to the level of the Lys fragment. The proportions of Hly, Lys, and deuterated Lys fragments are consistent with the structure shown in the paper by Tanzer *et al*. (4). Fragmentation of the *m*/*z* ion 575 from NaBD_4_ reduction (Fig. S6) clearly showed reduction occurring on the “core” and no core fragments without deuterium, which is consistent with the structure proposed by Tanzer *et al*. (4).

We concluded that the *m*/*z* 574 ion seen here is consistent with the proposed structure, and we proceeded on the basis that it was likely to be the same compound that Tanzer *et al.* (4) analyzed and called HHMD.

### Chemical synthesis

All nonlabelled reagents used were obtained from Sigma Aldrich and stable isotopically labelled compounds from CK Isotopes Ltd (UK). Solvents were obtained from Romil Ltd. (Cambridge, UK) and were either superpure or ultrapure grade. Mass spectra were run on Sciex QTRAP4000 and Micromass Quattro Ultima mass spectrometers. NMR spectra were run on a Bruker 500 MHz DCH Cryoprobe spectrometer in the University of Cambridge Chemistry Department.

#### 

##### Hx-Lys synthesis

The following synthesis produced an inseparable mixture of ε*N*-glycated lysine (by NMR) which looked the same as endogenous glycated lysine by MS. It is likely that some racemization occurred at chiral centers in this synthesis, and this accounts for some or all of the additional complexity seen in the NMR spectra. This synthesis enabled a ^13^C_6_ internal standard to be synthesized which behaved well in the MS assay.

*N*α-Benzyloxycarbonyl-l-lysine (200 mg, 1 eq) was added to glucose (1.55 g, 12 eq) in methanol (30 ml). The reaction mixture was refluxed for 4 h and then allowed to cool. Sodium borohydride (324 mg, 12 eq) was then added, and the reaction mixture was left to stir overnight. A 1 M concentration of hydrochloric acid (3 ml) was then added, and the solution was concentrated to an oil. Water (30 ml) was added and the pH adjusted to 4. The solution was loaded onto a 5-g C_18_ solid-phase extraction cartridge (BondElute; Varian) and washed with 5 mm HCl (20 ml), and the glycated materials were eluted with 10% acetonitrile. The solution was then freeze-dried. HPLC showed a number of peaks which were analyzed by MS. The largest peak had the correct mass of 445.5 (MH^+^). Starting with the crude glycated material (80 mg), pure monoglycated Z-Lys (14 mg) was isolated after 3 rounds of purification on a C_18_ Luna 10- × 250-mm column with a 5% to 50% B gradient (A, water–0.1% TFA; B, 90% acetonitrile–10% water–0.1% TFA).

Deoxyhexitolyl-Nα-benzyloxycarbonyl-l-lysine (10 mg) was then added to water (1 ml) with methanol (0.2 ml) and 10% Pd/C (3.8 mg). The reaction was placed under hydrogen at atmospheric pressure and left for 1.5 h. Completion of the reaction was checked by MS. The Pd/C was removed by filtering the reaction through a 3000-molecular-weight-cutoff filter and freeze-dried to give 4 mg of the desired product, 6-(2,3,4,5,6-pentahydroxyhexylamino)-2-aminohexanoic acid (Hx-Lys).

The retention time and fragmentation pattern matched that of endogenous glycation with a parent *m*/*z* of 311.18. NMR data are shown in Fig. S7, *a* to *f* (^1^H,^13^C, DEPT135, ^1^H COSY, heteronuclear single quantum coherence, and heteronuclear multiple-bond correlation). Fragmentation patterns are shown in Fig. S8.

This synthesis was repeated for both [^12^C_6_]glucose and [^13^C_6_]glucose. For the [^13^C_6_]glucose reaction, the quantities of the reagents were scaled down and a ratio of 1:1 [^13^C_6_]glucose:Z-Lys was used.

##### cHx-Lys synthesis

2,3,4,6-Tetrabenzyl glucose (1 g, 1 eq) was suspended in methanol (50 ml) and water (70 ml). 1,4-Dioxane (40 ml) was added, and the solution was warmed gently to 40°C. Sodium borohydride (210 mg, 3 eq) was added in 1 mm sodium hydroxide solution (1 ml). The reaction mixture was stirred overnight, but the reaction was found to be only 50% complete by TLC (toluene:ethyl acetate, 4:1). An additional portion of sodium borohydride (210 mg, 3 eq) was added and stirred until the reaction was complete (24 h). Hydrochloric acid (1 M; 3 ml) in water (10 ml) followed by EtOAc (60 ml) was added to the reaction mixture, and the organic layer was separated, dried over MgSO_4_, and concentrated under vacuum. The product was purified on silica with toluene:ethyl acetate (7:3) as the eluent to provide pure product, 2,3,4,6-tetrakis(benzyloxy)hexane-1,5-diol (745 mg, 75% yield). NMR spectra were in agreement with previously reported data (22). ESI MS gave *m*/*z* 543.27, MH^+^.

DMSO (105 μl, 8 eq) was added to CH_2_Cl_2_ (4 ml) and cooled to −78°C. A solution of trifluoroacetic anhydride (153 μl, 6 eq) in CH_2_Cl_2_ (1 ml) was added dropwise over 20 min and left to react for a further 10 min. 1,3,4,5-Tetrabenzyl(2*S*,3*R*,4*R*,5*R*)-hexane-1,2,3,4,5,6-hexol (100 mg, 1 eq) in CH_2_Cl_2_ (1 ml) was added dropwise over 15 min The reaction was stirred for 45 min, and then triethylamine (257 μl, 10 eq) was added over 10 min and left to react for additional 10 min. The reaction mixture was allowed to warm to room temperature over 30 min and left to stir for a further 1 h. Saturated brine was added, and the product was extracted into CH_2_Cl_2_, dried over MgSO_4_, and concentrated. The crude dicarbonyl product was taken on to the next step without purification.

NaBH_3_CN (46.8 mg, 4 eq) and Na_2_SO_3_ (93 mg, 4 eq) were added to the stirred solution of *N*α-benzyloxycarbonyl-l-lysine benzyl ester (82.5 mg, 1.18 eq) in methanol (0.5 ml). The reaction was cooled in the ice bath and stirred for 10 min, and then the dicarbonyl product (prepared above) was added as methanol solution (1.5 ml). After 20 min the reaction mixture was warmed to room temperature and stirred for 20 h. The solvent was evaporated, and the residue was dissolved in dichloromethane and washed with 1 M sodium bicarbonate solution. The organic phase was dried over MgSO_4_ and concentrated under vacuum at room temperature. The crude product was purified on silica with toluene:ethyl acetate (95:5, with 0.5% Et_3_N added) as the eluent followed by crystallization from methanol to afford benzyl-1-((benzyloxy)carbonyl)-5-(3,4,5-trihydroxy-2-(hydroxymethyl)piperidin-1-yl)pentyl carbamate as a white powder (8 mg, 5% yield). ^1^H NMR data are shown in Fig. S9. ESI MS gave *m*/*z* 877.52 MH^+^.

Benzyl-1-((benzyloxy)carbonyl)-5-(3,4,5-trihydroxy-2-(hydroxymethyl)piperidin-1-yl)-pentyl carbamate (7 mg, 1 eq) was dissolved in ethyl acetate (0.5 ml), followed by addition of methanol:water (1:1, 2 ml). Hydrochloric acid (1 M; 27 μl, 3.4 eq) was added followed by 10% Pd/C (20 mg). The reaction was stirred under hydrogen at atmospheric pressure, and progress was monitored by MS. After 96 h, the reaction mixture was filtered through a Costar 0.22-μm spin filter to remove the Pd/C and concentrated under reduced pressure. The product was freeze-dried from water to afford 2-amino-6-(3,4,5-trihydroxy-2-(hydroxymethyl)piperiif din-1-yl)hexanoic acid (cHx-Lys) (2.6 mg, 98% yield). NMR data are shown in Fig. S10, *a* to *e* (^1^H, double quantum-filtered COSY, total correlation spectroscopy, ^13^C, and heteronuclear single quantum coherence). ESI MS gave *m*/*z* 293.2 MH^+^.

### Physical testing

#### 

##### Stress-strain profile measurements

Physical testing was undertaken using a Microtest 200N tensile stress stage (Deben UK Ltd.) fitted with a 20-N load cell and a petri dish bath to allow immersion of the sample. With a distance of 14 mm between the jaws, individual processed fibers were clamped submerged in PBS (pH 7.4) at room temperature and preloaded to a force of 0.01 N. The diameter of each fiber was assessed along its length and the smallest dimension used to calculate stress. Force was measured as the fiber was taken to breaking point at a speed of 1 mm/min.

### Statistical analysis

When 2 groups were compared, ratio-paired Student *t* tests were used. When more than 2 groups were compared, one-way ANOVA followed by Tukey's multiple comparisons tests were applied. Linear and nonlinear regressions were applied to quantify the association between continuous variables. Significance was defined as a *p* value of <0.05, and *p* values are reported in the figures. Data analysis was performed using GraphPad Prism 8.

## Data availability

All data are available from the corresponding author upon request.

## Supplementary Material

Supporting Information
