# Supplementary material for: Age-related changes in the physical properties, cross-linking, and glycation of collagen from mouse tail tendon
Source: J Biol Chem. 2020 May 7;295(31):10562–71. doi: 10.1074/jbc.RA119.011031 (PMC7397091; doi:10.1074/jbc.RA119.011031)
Supplement: Supporting Information [file supp_295_31_10562__index.html]

Age-related changes in the physical properties, cross-linking, and glycation of collagen from mouse tail tendon — Changes in collagen crosslinks and glycation with age — Age-related changes in the physical properties, cross-linking, and glycation of collagen from mouse tail tendon — Changes in collagen crosslinks and glycation with age — Supporting Information 

# Age-related changes in the physical properties, cross-linking, and glycation of collagen from mouse tail tendon

## Supporting Information

- Supporting Information for changes in collagen crosslinking and glycation with age - Supplimantary Figures
