# Supplementary material for: Age-related changes in the physical properties, cross-linking, and glycation of collagen from mouse tail tendon
Source: J Biol Chem. 2020 May 7;295(31):10562–71. doi: 10.1074/jbc.RA119.011031 (PMC7397091; doi:10.1074/jbc.RA119.011031)
Supplement: Supporting Information [file supp_RA119.011031_155781_4_supp_524005_q9ss7z.pdf]

Supplementary figures follow:

Figure S1

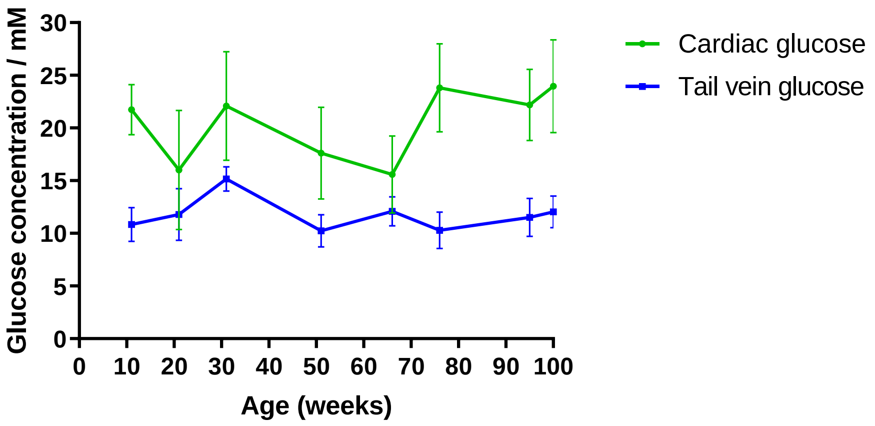

Figure S1a Graphs showing the measured glucose levels in C57Bl/6 mice (mean $\pm SD$) after fasting for 6 hours in different age groups (n=5-12) immediately after culling.

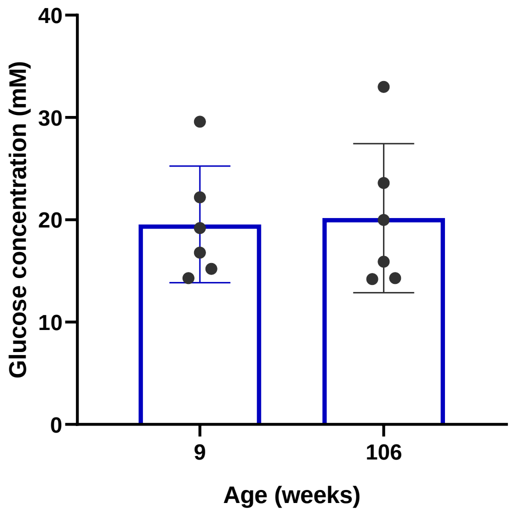

Figure S1b Graphs showing the measured cardiac glucose levels in non-fasting C57Bl/6 mice in different age groups (n=6) immediately after culling (mean $\pm SD$).

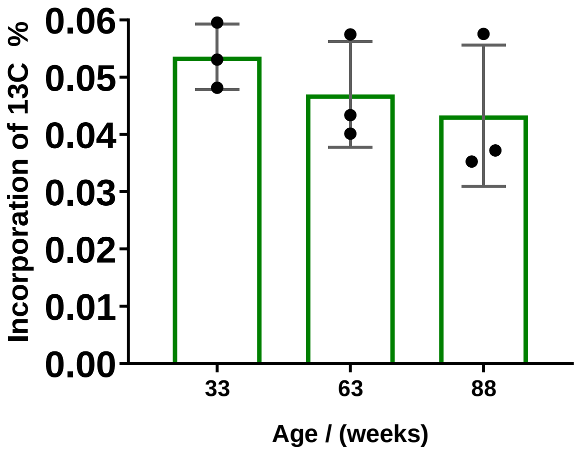

Figure S1c The incorporation of $^{13}C_6$ Lysine into tendon fibres $per\ day$ (mean $\pm SD$). Mice were fed $^{13}C_6$ Lysine for 28 days (n=3).

Figure S2

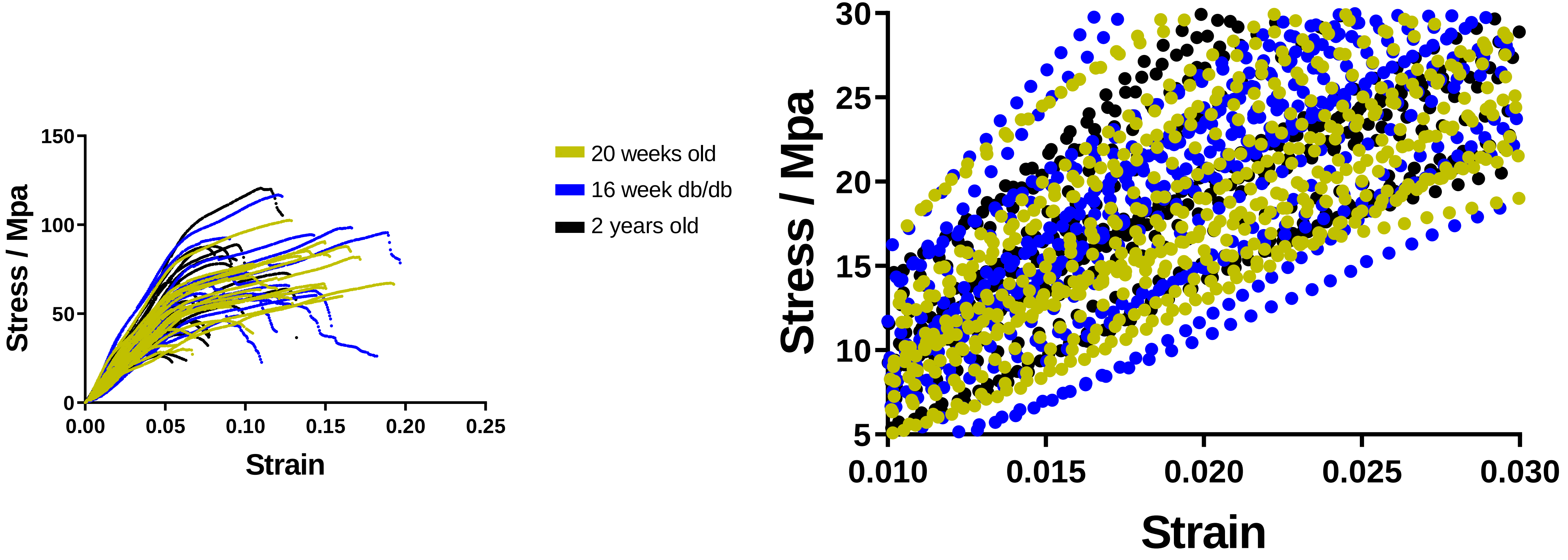

Figure S5 Overlay of traces in figure 2 and 4a (main text) and expansion. The steeper lines to the left show increased stiffness.

Figure S3

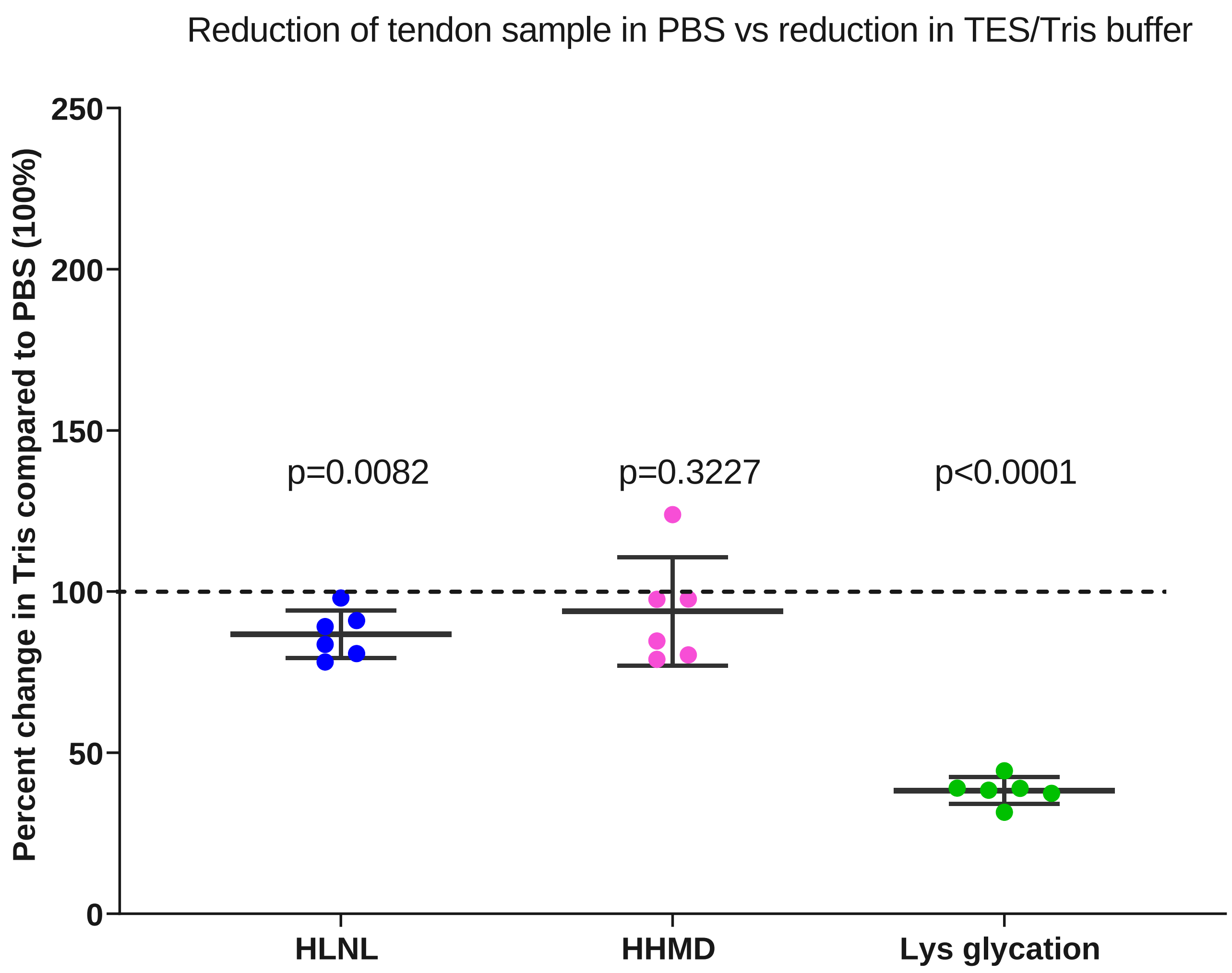

Reduction of tendon sample in PBS vs reduction in TES/Tris buffer
Percent change in Tris compared to PBS (100%)
250
200
150
100
50
0
p=0.0082
p=0.3227
p<0.0001
HLNL
HHMD
Lys glycation

Figure S4

Fragmentation of $NaBH_4$ reduced HHMD signal

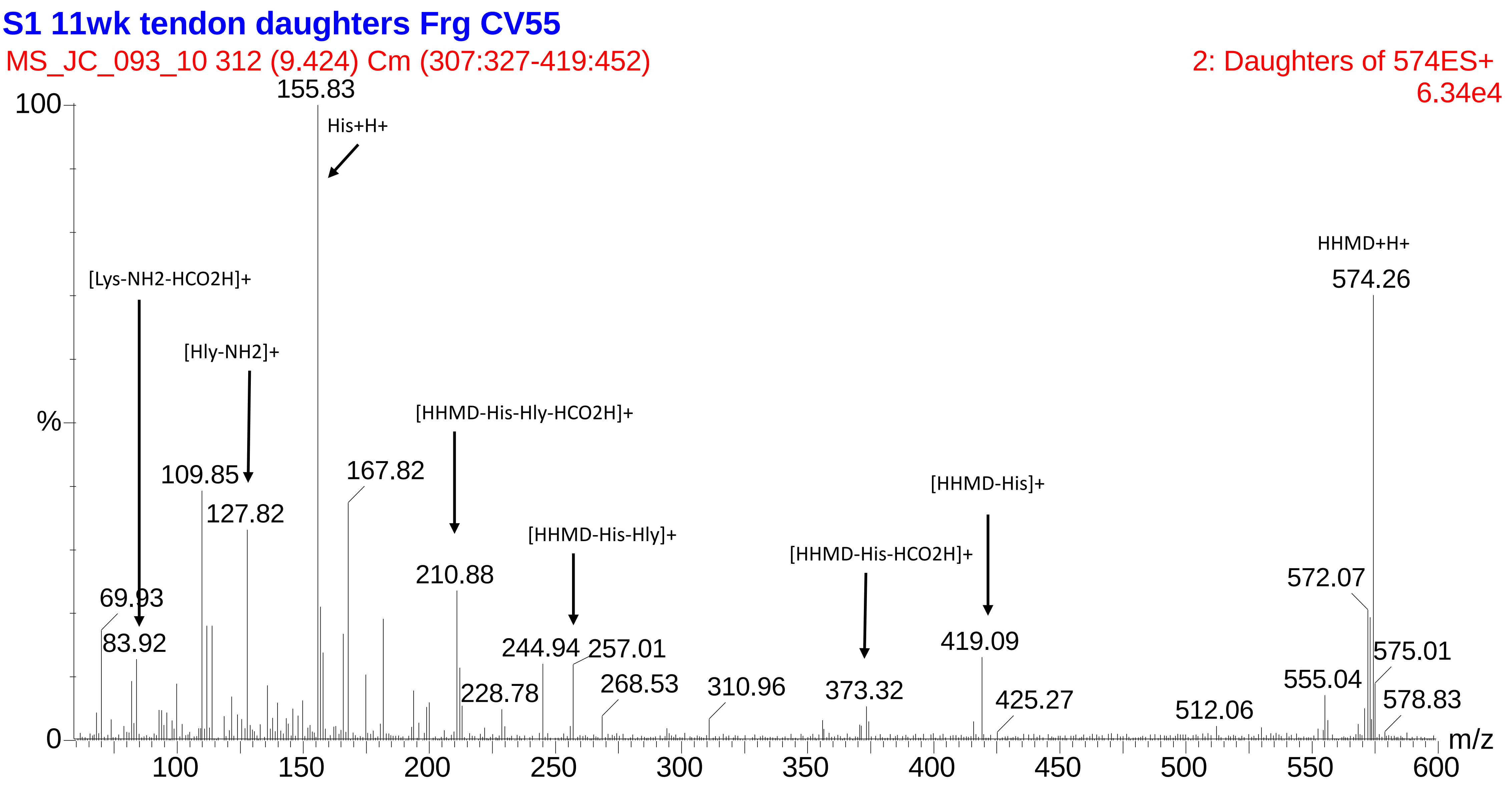

Figure S5 Reduction of HHMD with $NaBH_4$ and $NaBD_4$

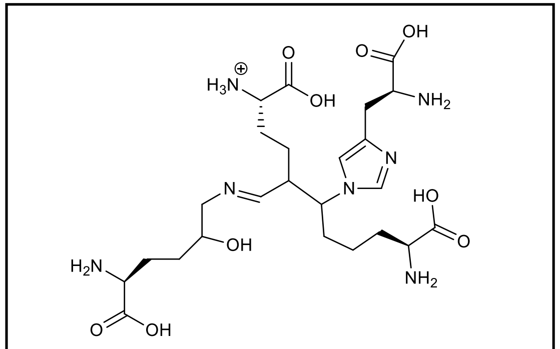

Un-reduced HHMD literature structure

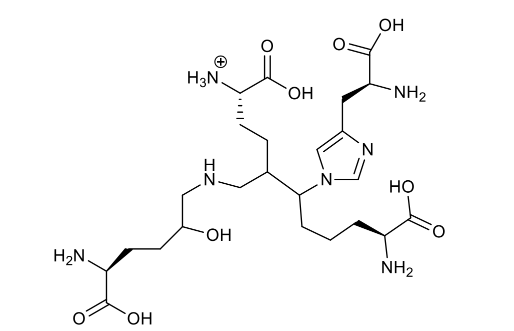

Reduced HHMD literature structure

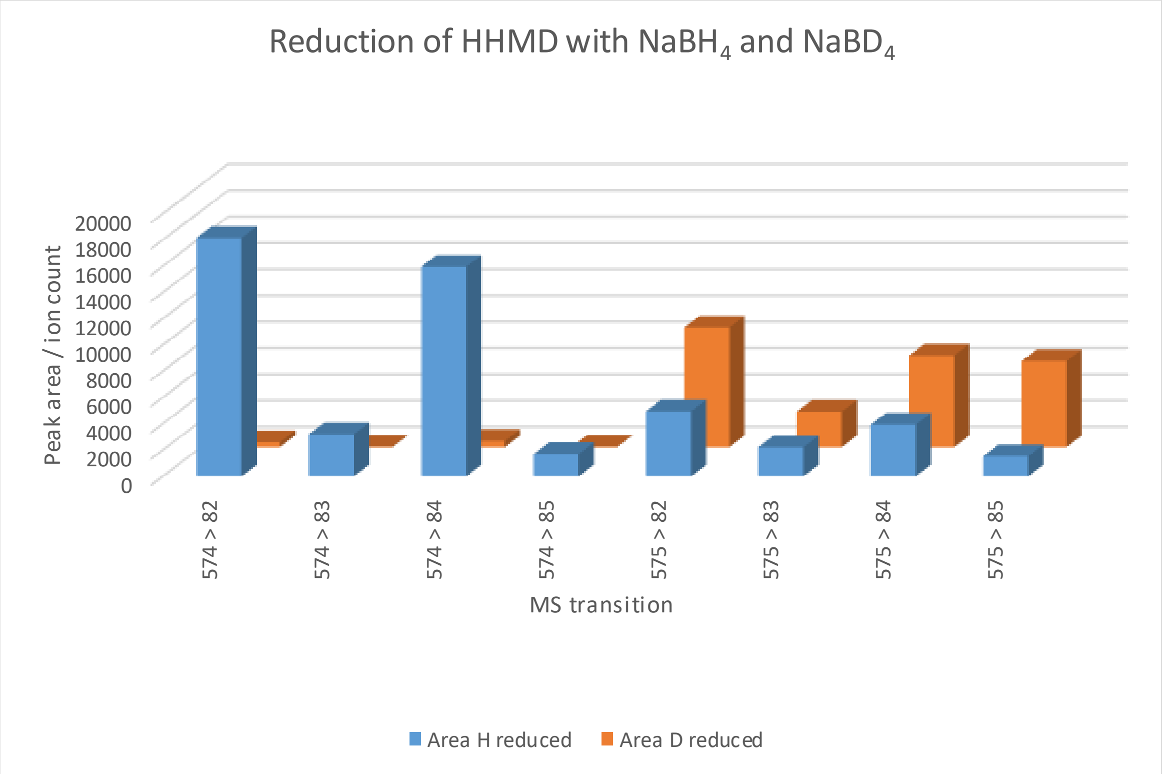

Interpretation of ion transition

Blue 574 > 82 indicates presence of

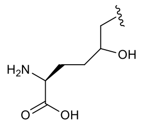

Blue 574 > 83 shows Lys fragment, protonated fragment or coincidental mass

Blue 574 > 84 indicates presence of

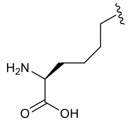

Blue 574 > 85 shows unidentified fragment or coincidental mass

Blue 575 transitions show 13C isotope natural abundance

Orange 575 > 82 indicates presence of

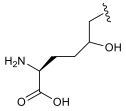

Orange 575 > 83 indicates presence of
(Higher than the expected
background signal)

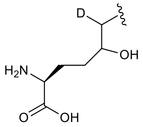

Orange 575 > 84 indicates presence of

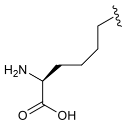

Orange 575 > 85 indicates presence of

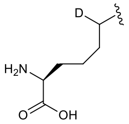

Figure S6

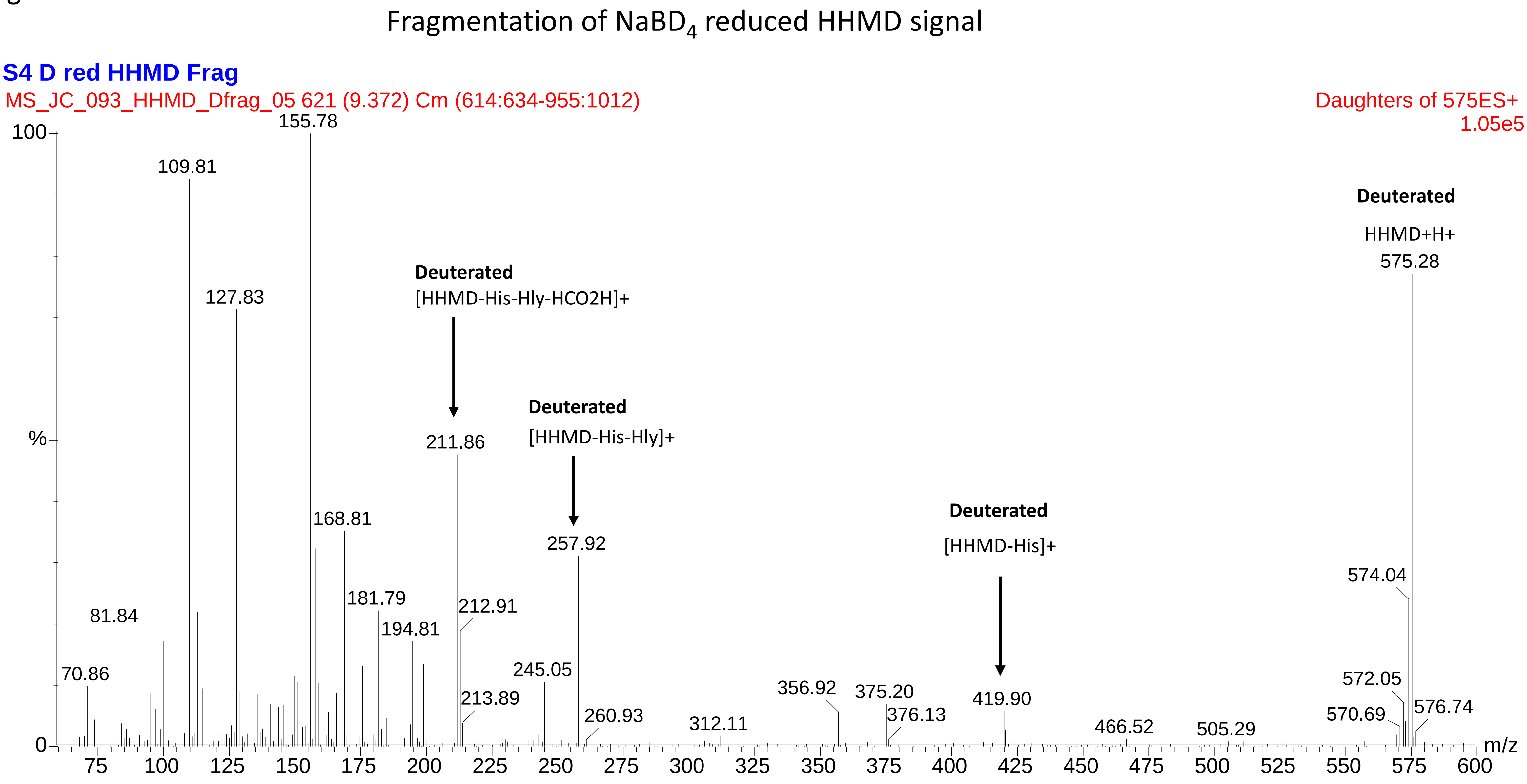

Fragmentation of NaBD$_4$ reduced HHMD signal
S4 D red HHMD Frag
MS_JC_093_HHMD_Dfrag_05 621 (9.372) Cm (614:634-955:1012)
Daughters of 575ES+
1.05e5
Deuterated
[HHMD-His-Hly-HCO2H]+
Deuterated
[HHMD-His-Hly]+
Deuterated
[HHMD-His]+
Deuterated
HHMD+H+
155.78
109.81
127.83
168.81
181.79
194.81
81.84
70.86
211.86
212.91
213.89
257.92
245.05
260.93
312.11
356.92
375.20
376.13
419.90
466.52
505.29
575.28
574.04
572.05
570.69
576.74
100
%
0
m/z
75
100
125
150
175
200
225
250
275
300
325
350
375
400
425
450
475
500
525
550
575
600

Figure S7a

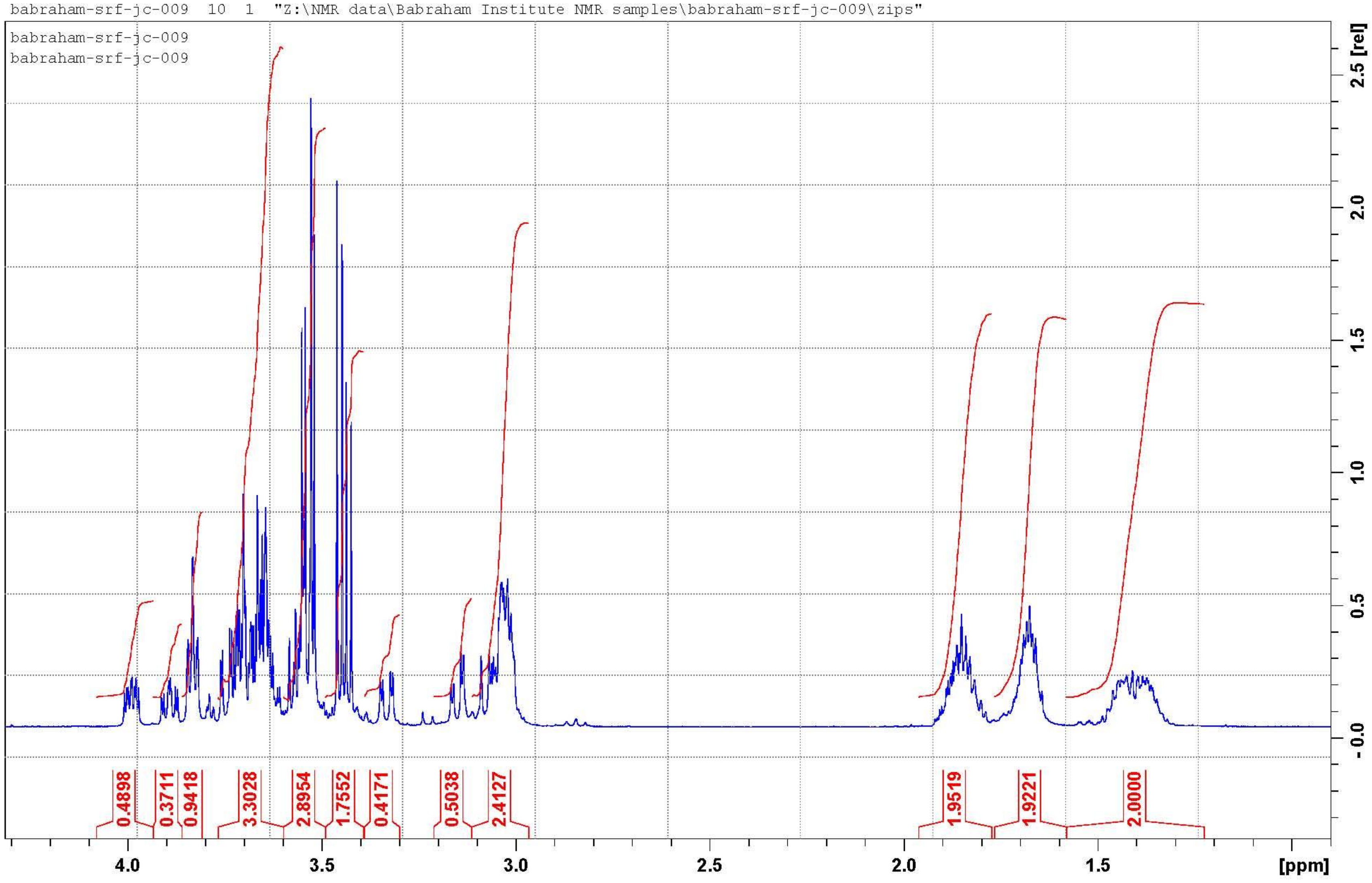

Proton NMR spectrum of Lys-Hx

Figure S7b

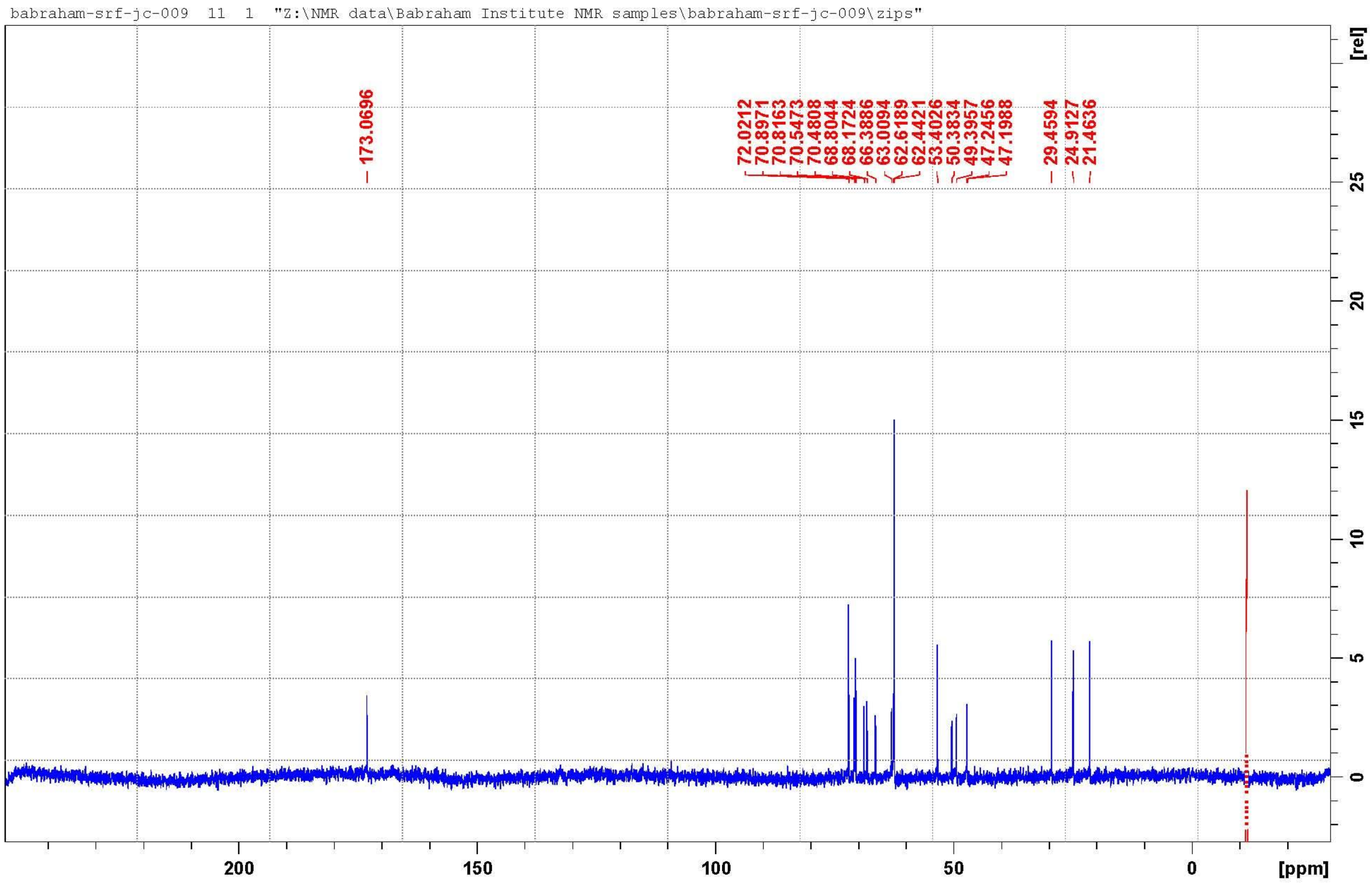

CPD carbon NMR spectrum of Lys-Hx

Figure S7c

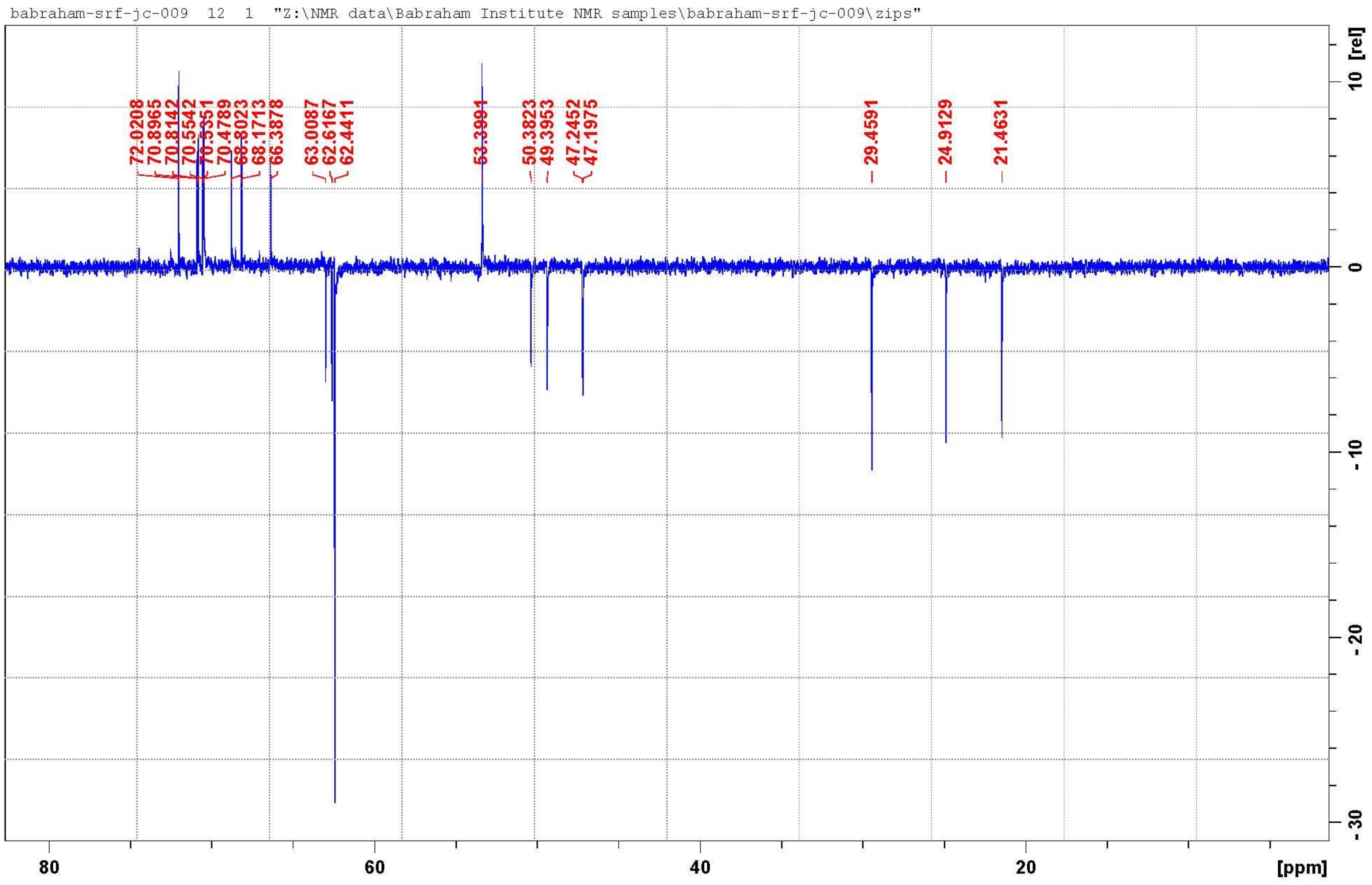

DEPT135 NMR spectrum of Lys-Hx

Figure S7d

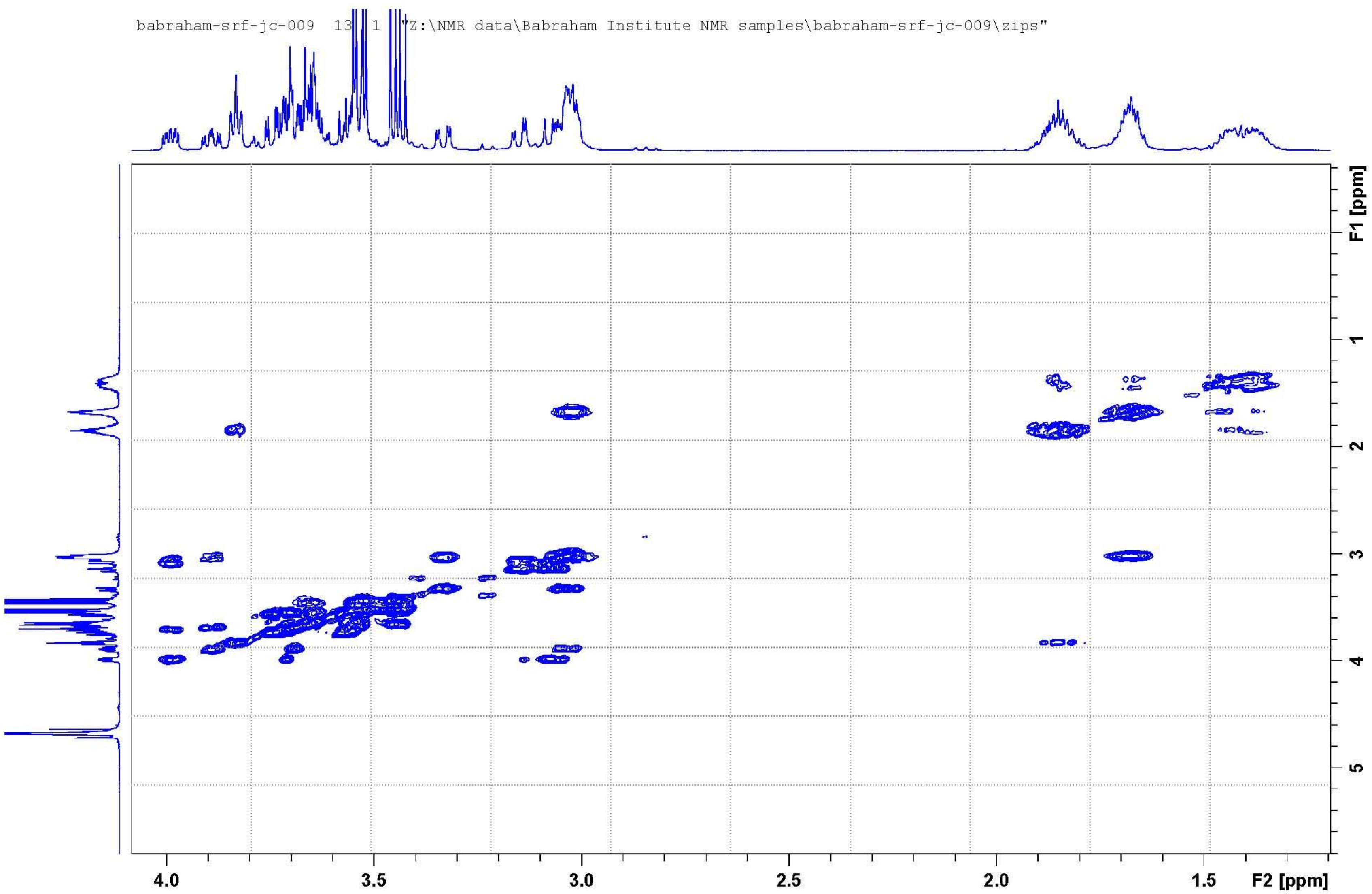

1H COSY NMR spectrum of Lys-Hx

Figure S7e

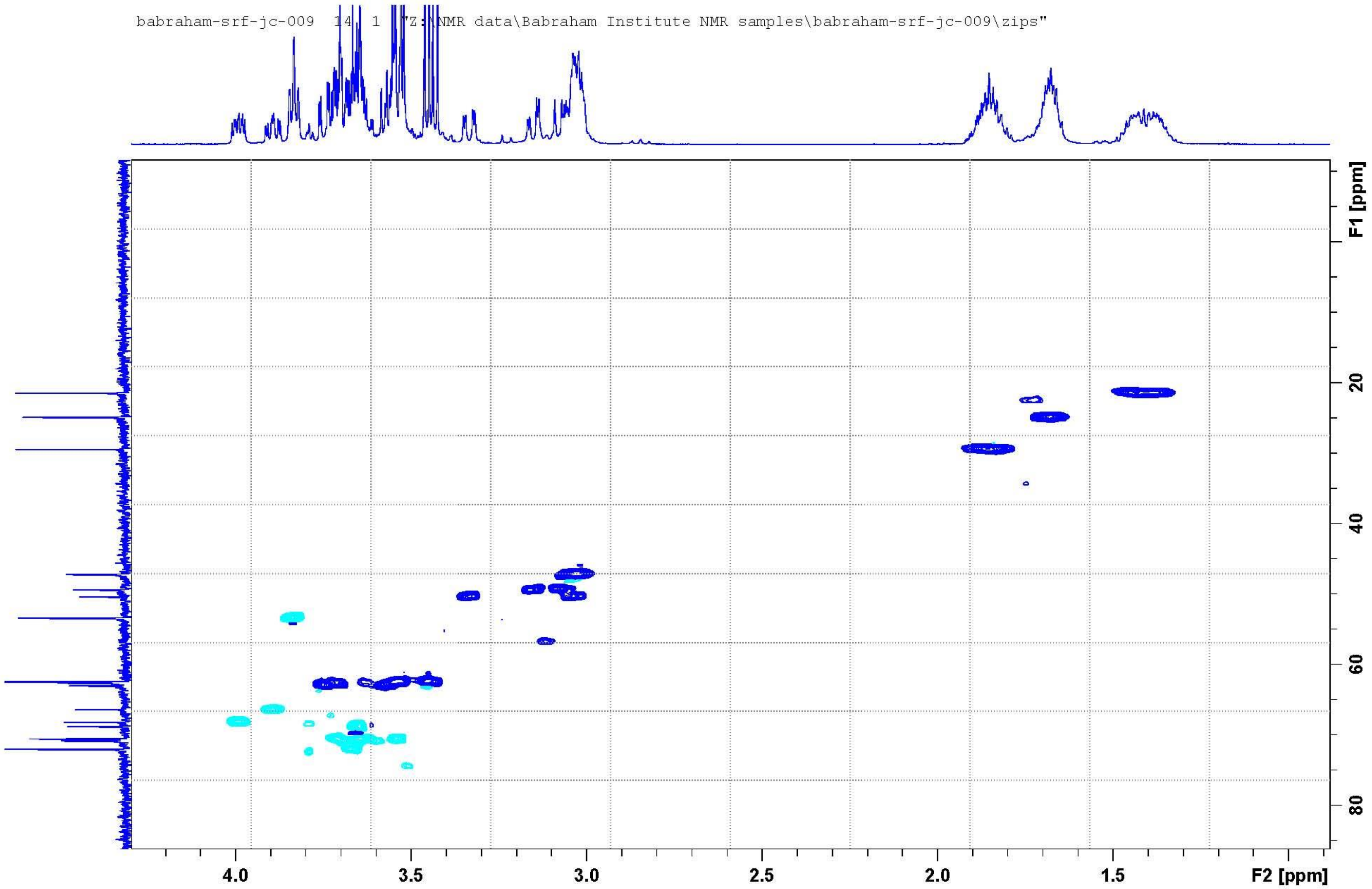

HSQC NMR spectrum of Lys-Hx

Figure S7f

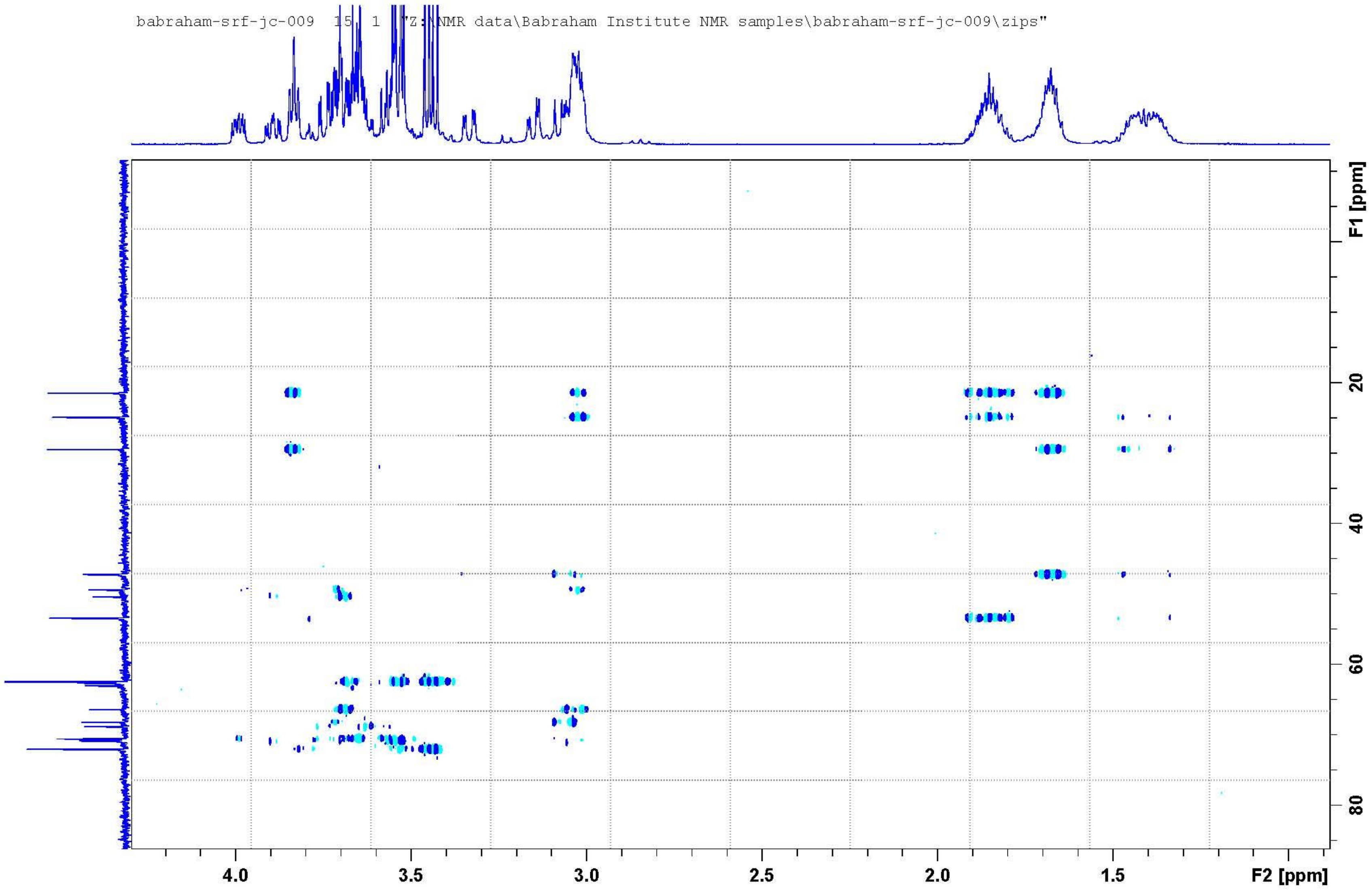

HMBC NMR spectrum of Lys-Hx

Figure S8

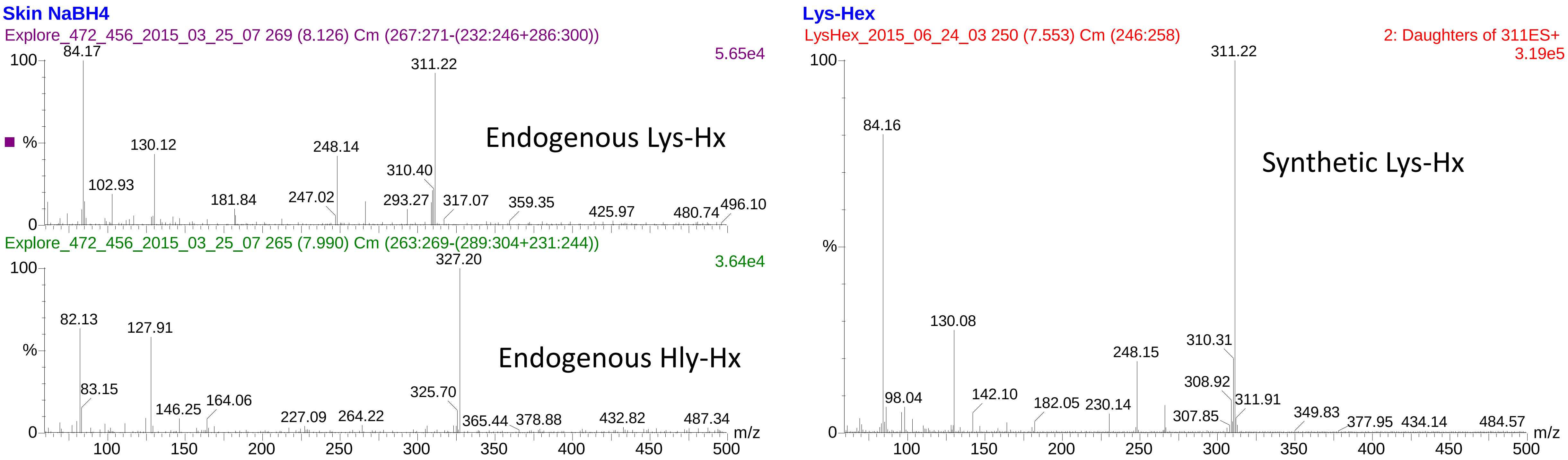

Fragmentation spectra of endogenous Lys-Hx from skin collagen and synthetic Lys-Hx

Figure S9

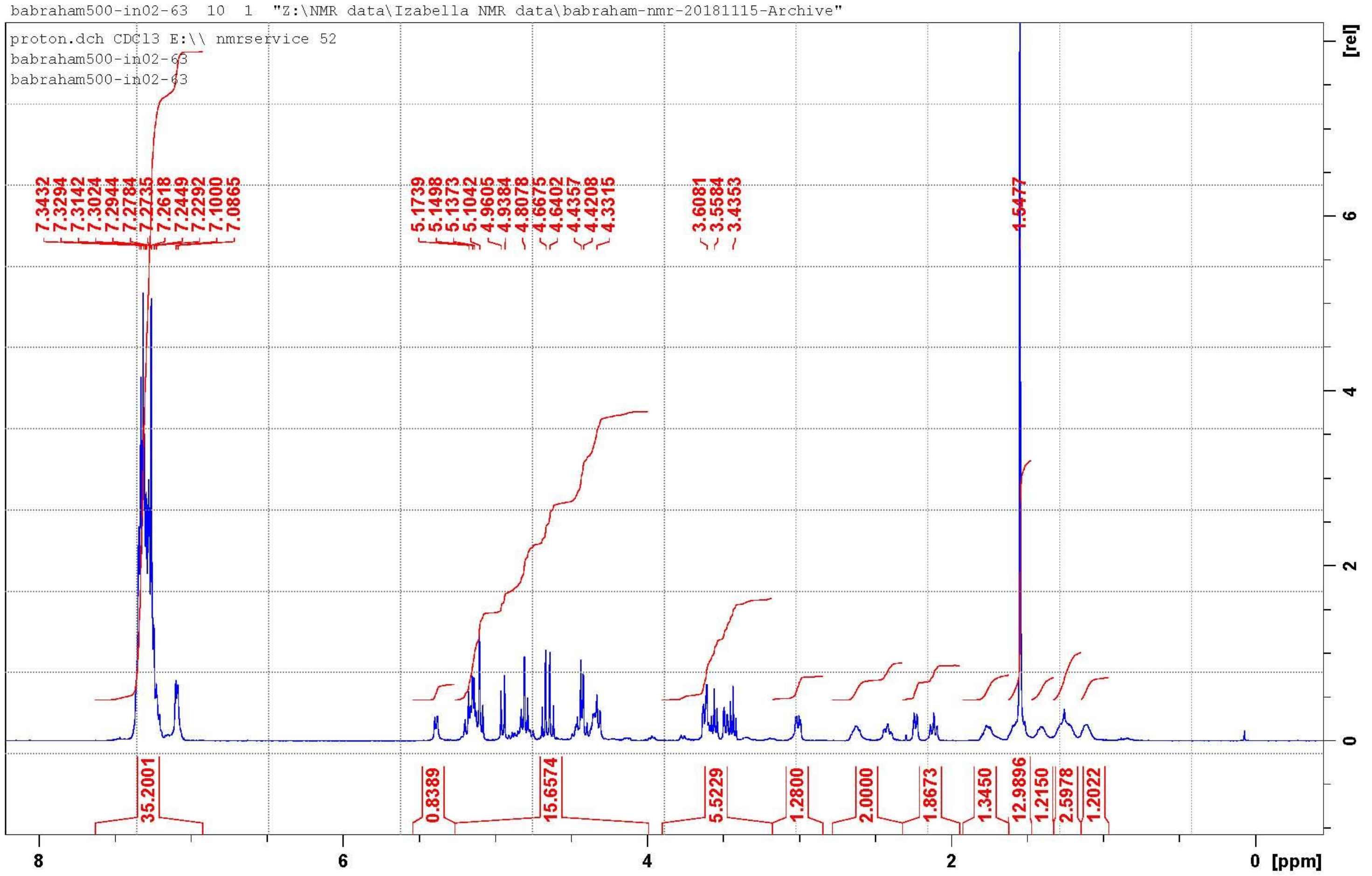

Proton NMR spectrum of benzyl-1-((benzyloxy)carbonyl)-5-(3,4,5-trihydroxy-2-(hydroxymethyl)piperidin-1-yl)pentyl carbamate

Figure S10a

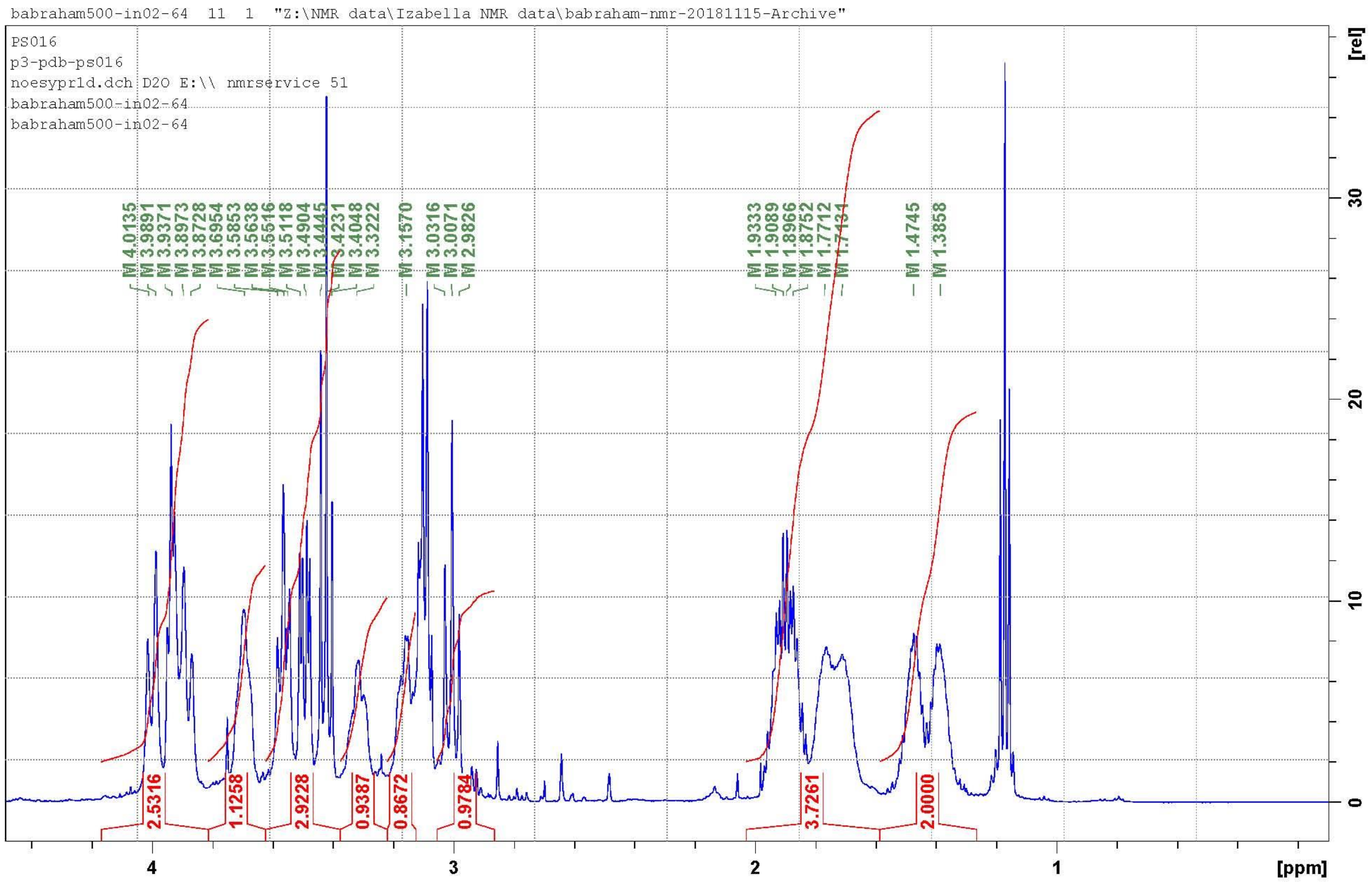

Proton NMR spectrum of 2-amino-6-(3,4,5-trihydroxy-2-(hydroxymethyl)piperidin-1-yl)hexanoic acid triethylamine salt (**Lys-cHx**)

Figure S10b

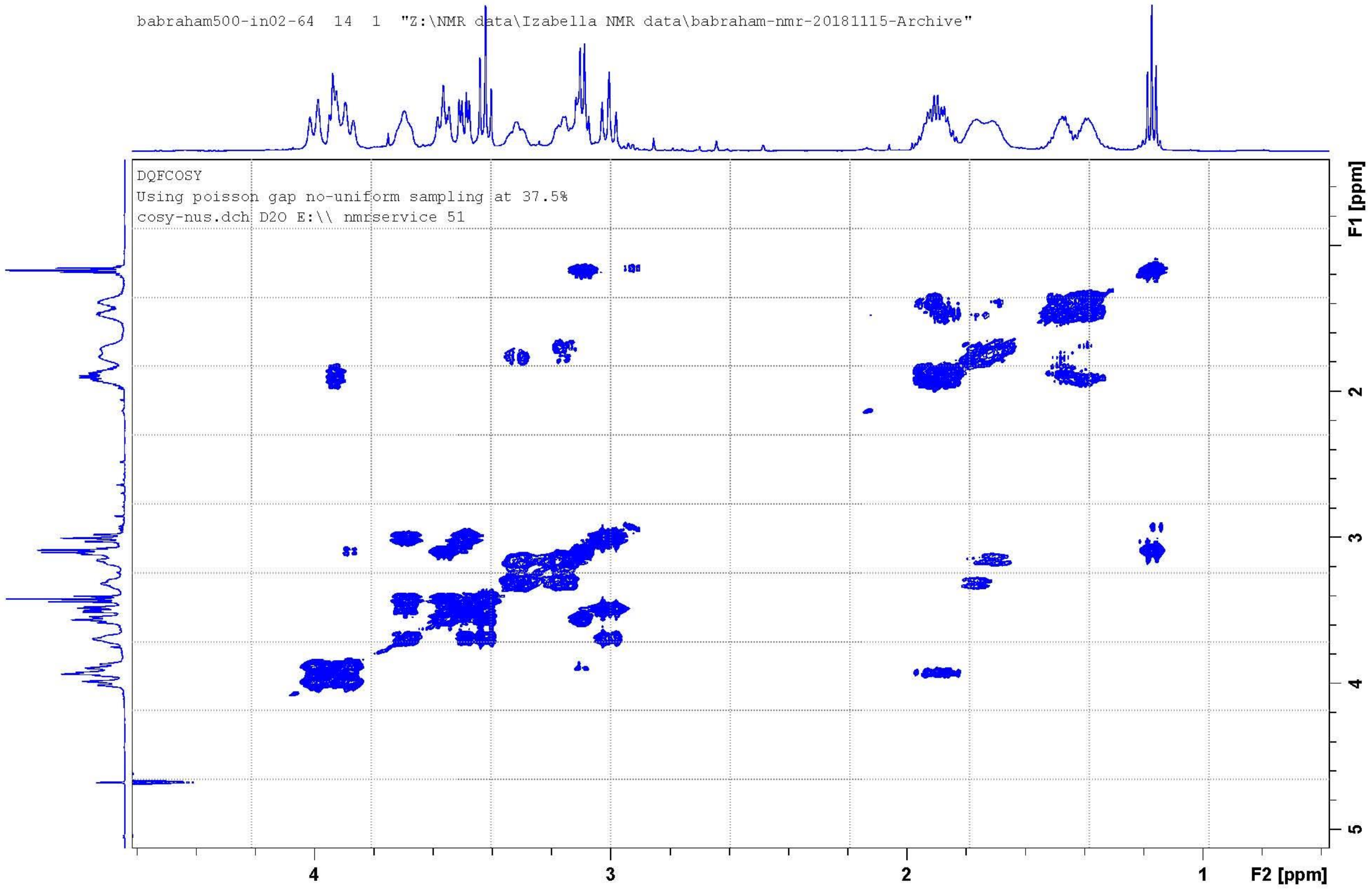

DQF COSY NMR spectrum of 2-amino-6-(3,4,5-trihydroxy-2-(hydroxymethyl)piperidin-1-yl)hexanoic acid triethylamine salt (**Lys-cHx**)

Figure S10c

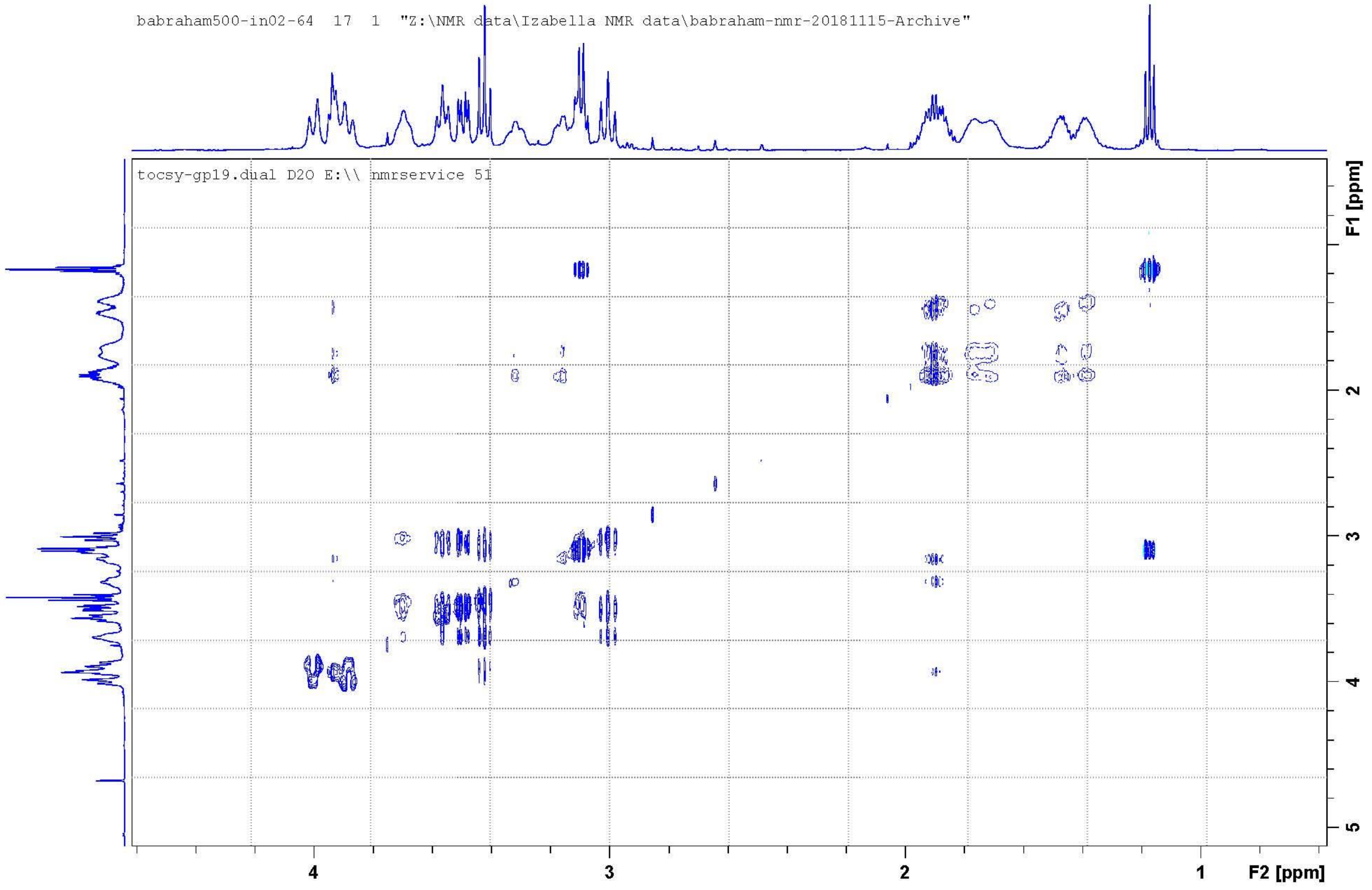

TOCSY NMR spectrum of 2-amino-6-(3,4,5-trihydroxy-2-(hydroxymethyl)piperidin-1-yl)hexanoic acid triethylamine salt (**Lys-cHx**)

Figure S10d

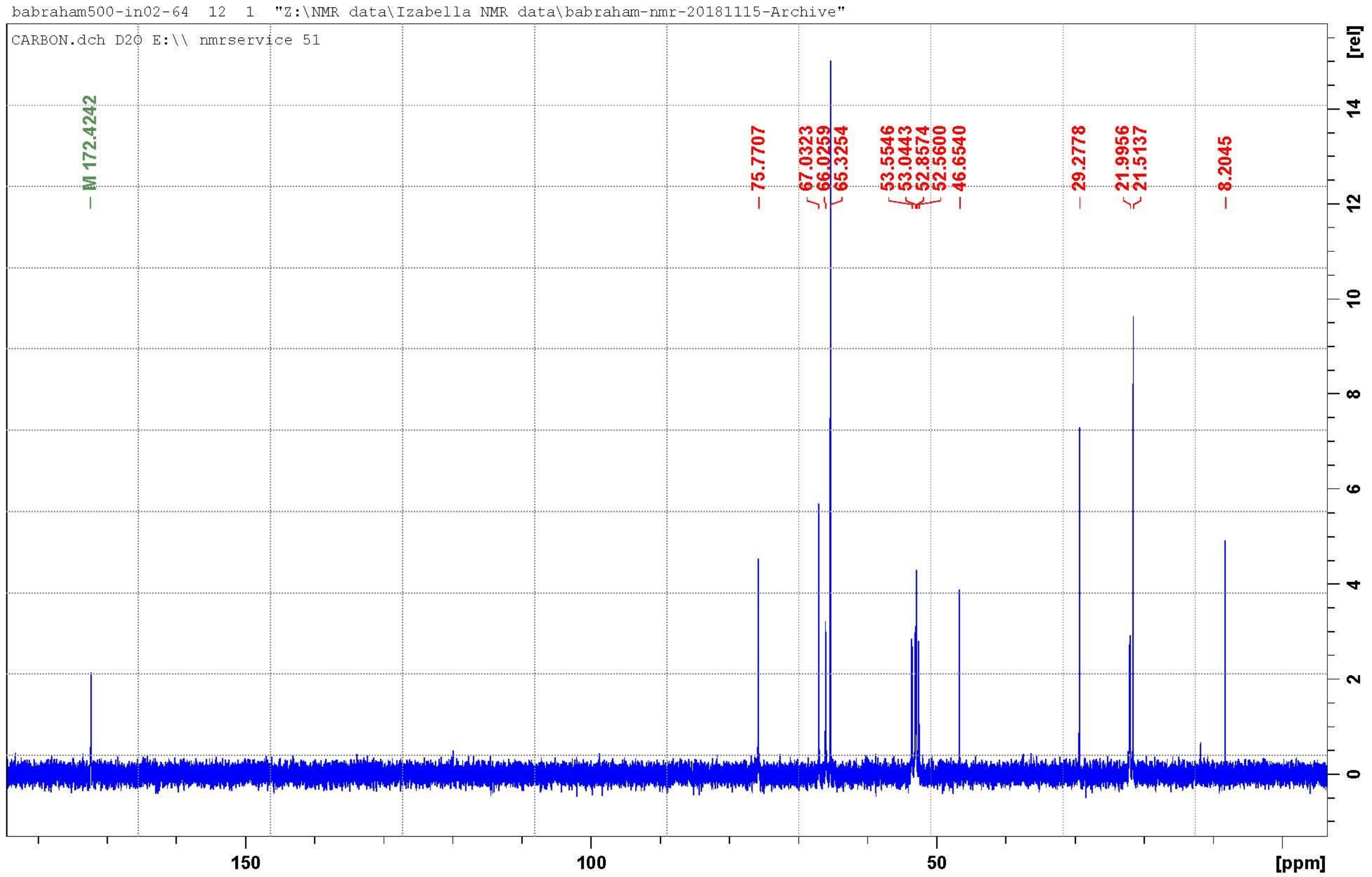

Carbon NMR spectrum of 2-amino-6-(3,4,5-trihydroxy-2-(hydroxymethyl)piperidin-1-yl)hexanoic acid triethylamine salt (**Lys-cHx**)

Figure S10e

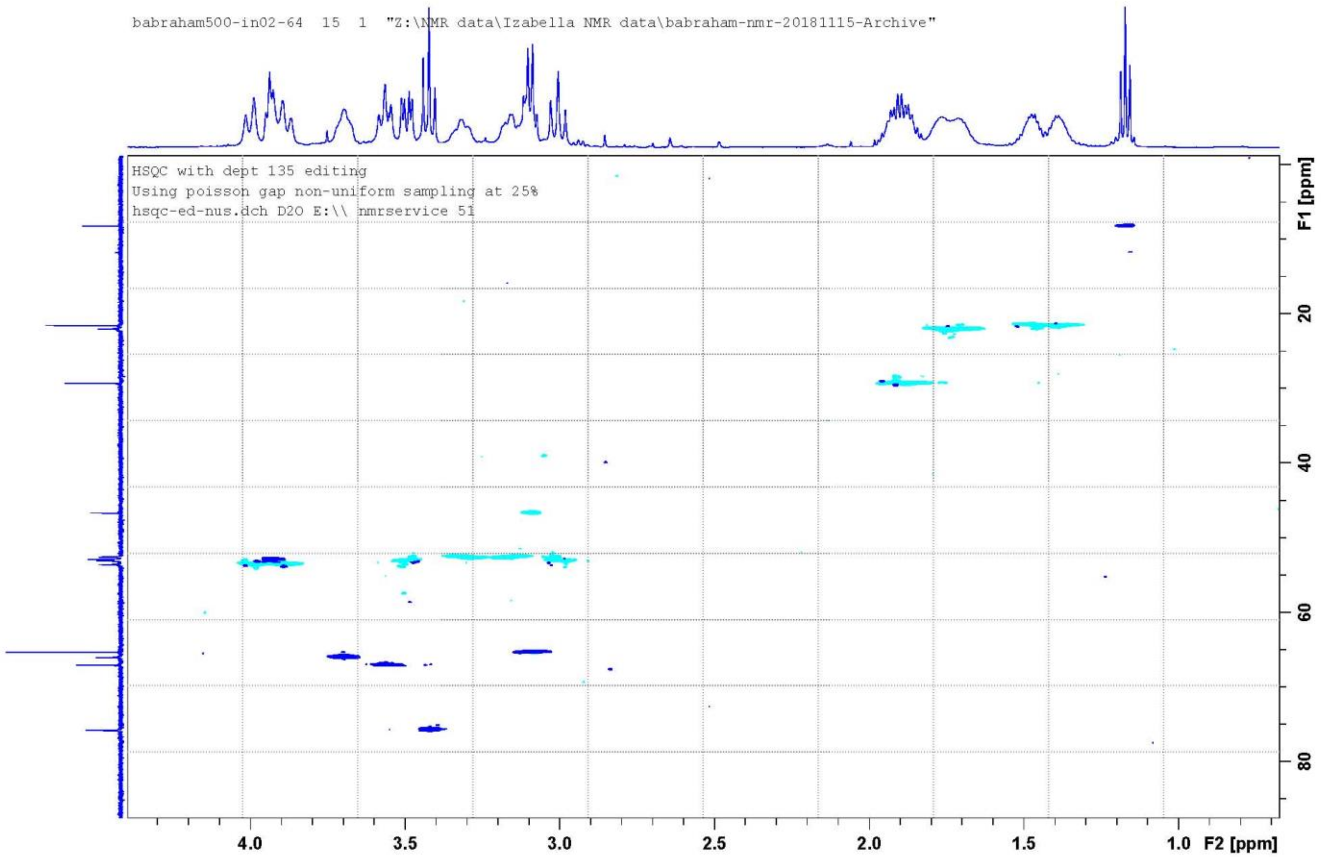

HSQC NMR spectrum of 2-amino-6-(3,4,5-trihydroxy-2-(hydroxymethyl)piperidin-1-yl)hexanoic acid triethylamine salt (**Lys-cHx**)
